# Supplementary figures and images for: mm2-ivh: simple and precise overlap detection in alpha satellite HORs with interval hashing (part 1 of 2)
Source: Bioinformatics. 2025 Dec 3;42(1):btaf648. doi: 10.1093/bioinformatics/btaf648 (PMC12766915; doi:10.1093/bioinformatics/btaf648)

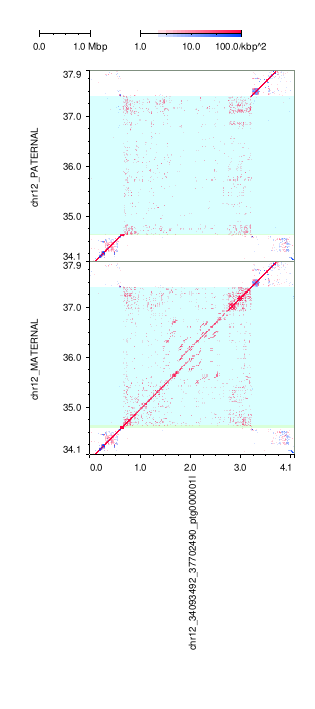

Supplement: btaf648_Supplementary_Data [file btaf648_supplementary_data.zip › Supplementary_Data/supplementary_figures/main_experiment/chr12/hifiasm-ont-r1.png]

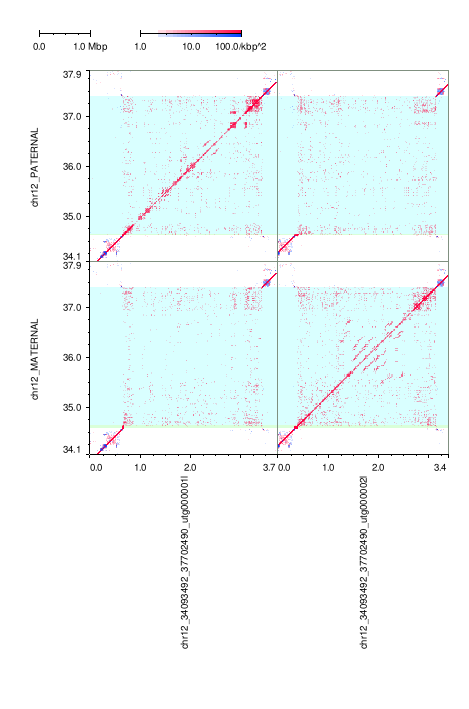

Supplement: btaf648_Supplementary_Data [file btaf648_supplementary_data.zip › Supplementary_Data/supplementary_figures/main_experiment/chr12/mm2-ivh-hpc.png]

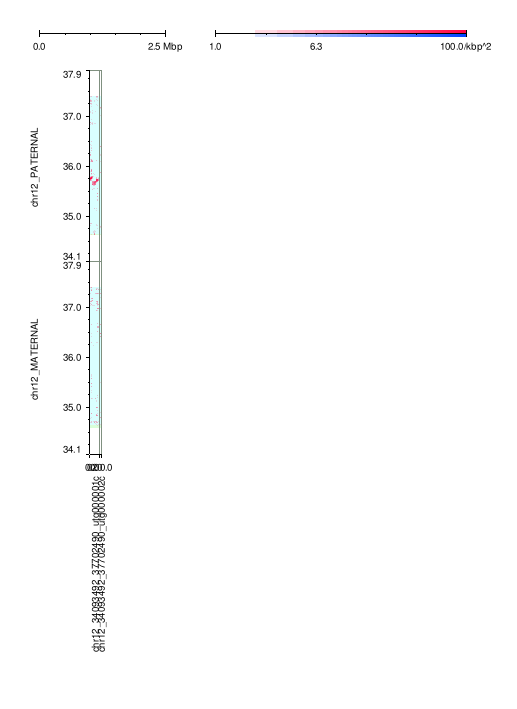

Supplement: btaf648_Supplementary_Data [file btaf648_supplementary_data.zip › Supplementary_Data/supplementary_figures/main_experiment/chr12/mm2-k133-hpc.png]

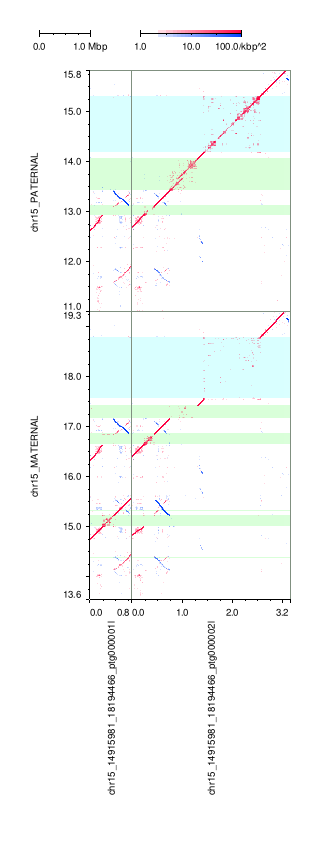

Supplement: btaf648_Supplementary_Data [file btaf648_supplementary_data.zip › Supplementary_Data/supplementary_figures/main_experiment/chr15/hifiasm-ont-r1.png]

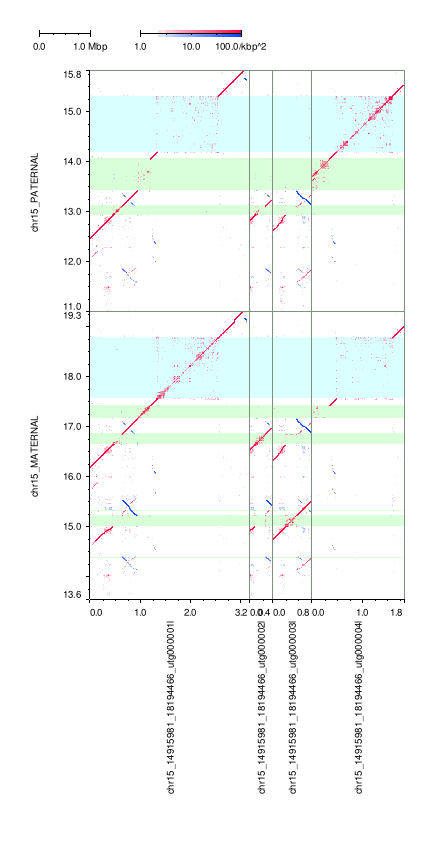

Supplement: btaf648_Supplementary_Data [file btaf648_supplementary_data.zip › Supplementary_Data/supplementary_figures/main_experiment/chr15/mm2-ivh-hpc.png]

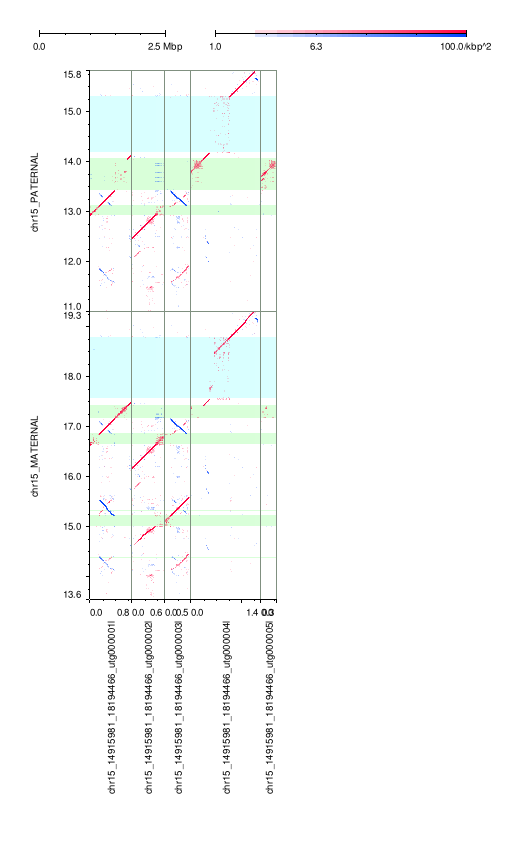

Supplement: btaf648_Supplementary_Data [file btaf648_supplementary_data.zip › Supplementary_Data/supplementary_figures/main_experiment/chr15/mm2-k133-hpc.png]

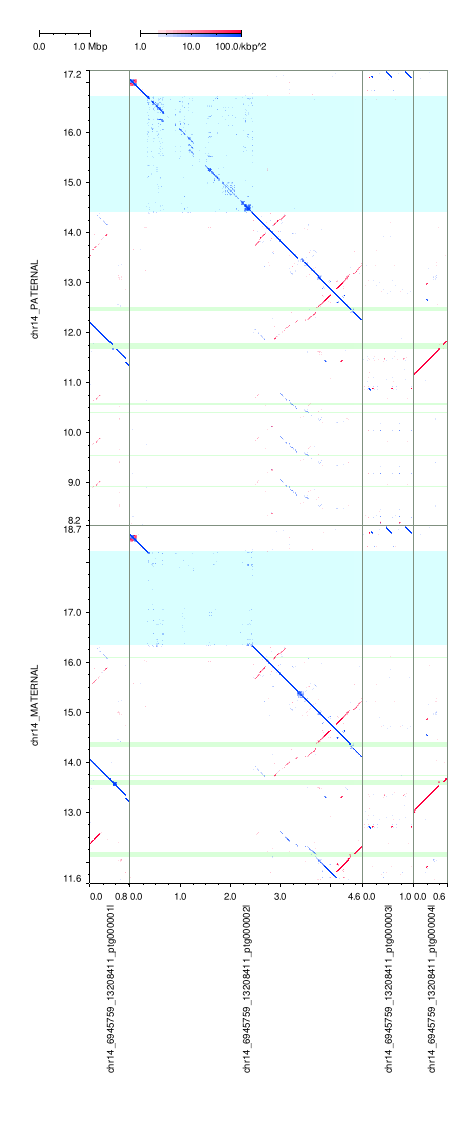

Supplement: btaf648_Supplementary_Data [file btaf648_supplementary_data.zip › Supplementary_Data/supplementary_figures/main_experiment/chr14/hifiasm-ont-r1.png]

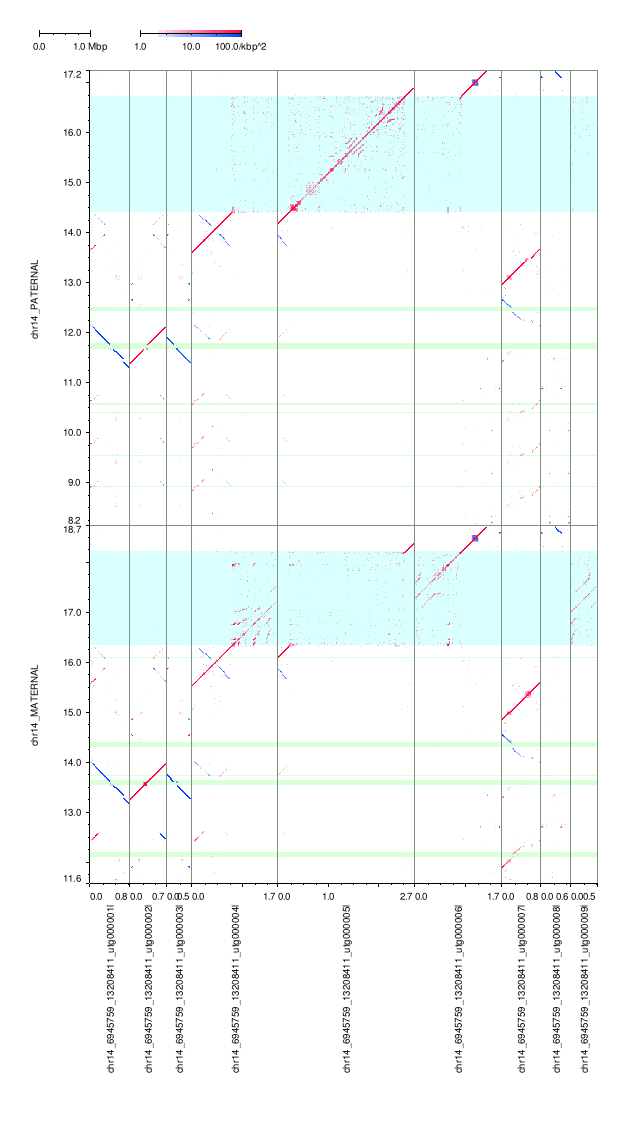

Supplement: btaf648_Supplementary_Data [file btaf648_supplementary_data.zip › Supplementary_Data/supplementary_figures/main_experiment/chr14/mm2-ivh-hpc.png]

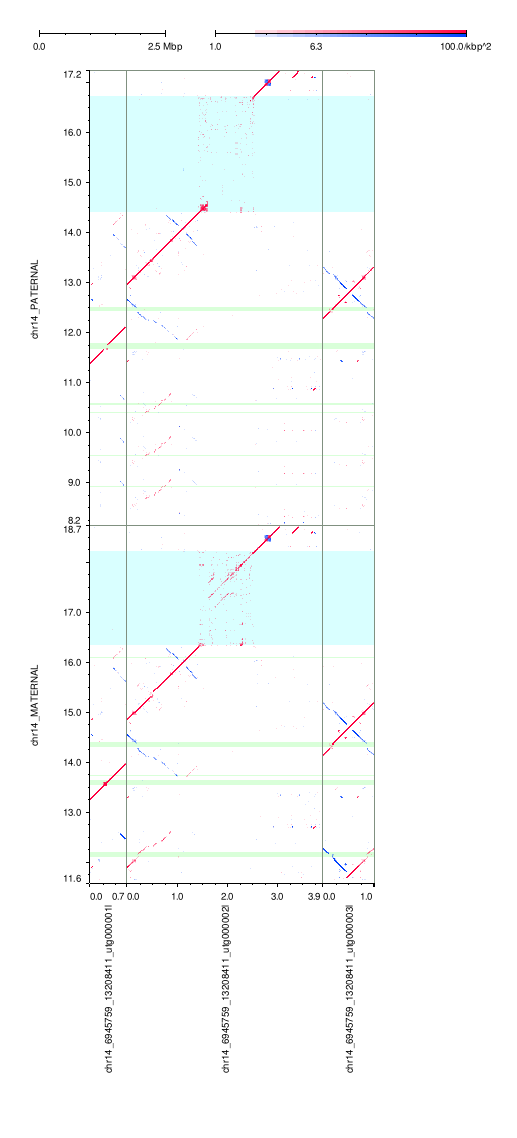

Supplement: btaf648_Supplementary_Data [file btaf648_supplementary_data.zip › Supplementary_Data/supplementary_figures/main_experiment/chr14/mm2-k133-hpc.png]

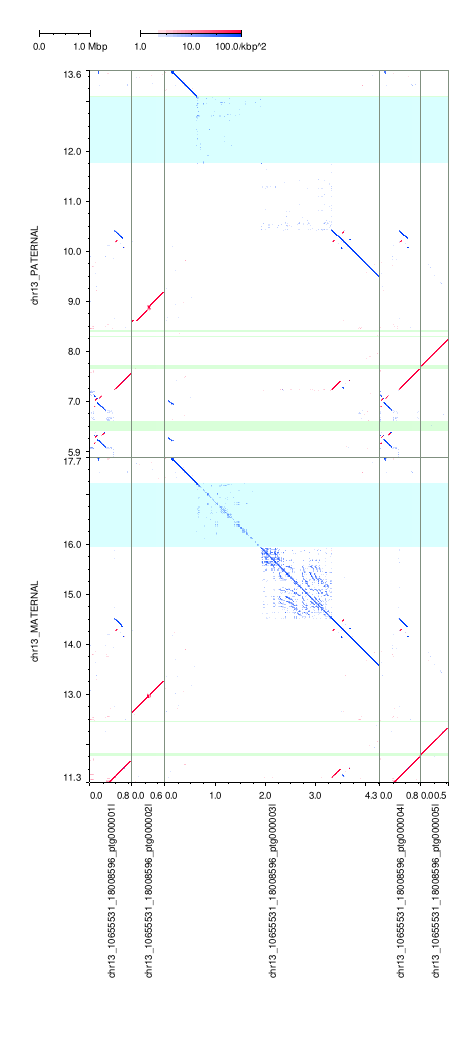

Supplement: btaf648_Supplementary_Data [file btaf648_supplementary_data.zip › Supplementary_Data/supplementary_figures/main_experiment/chr13/hifiasm-ont-r1.png]

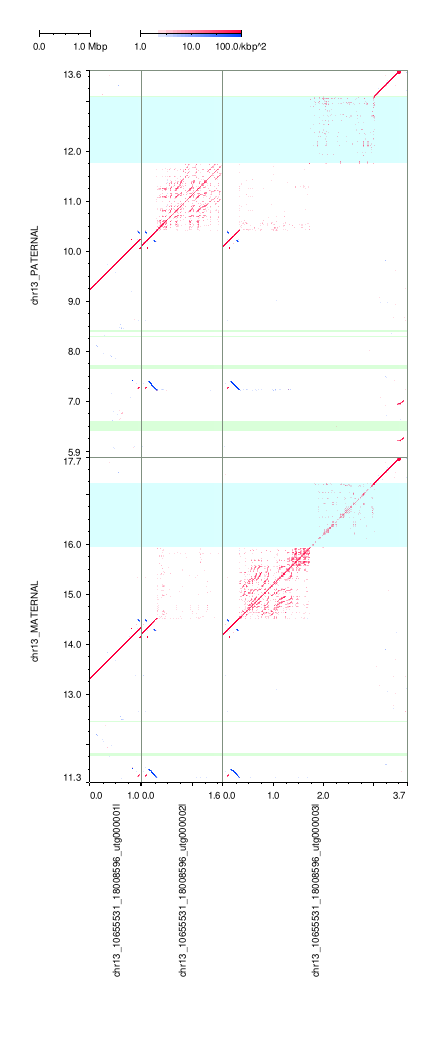

Supplement: btaf648_Supplementary_Data [file btaf648_supplementary_data.zip › Supplementary_Data/supplementary_figures/main_experiment/chr13/mm2-ivh-hpc.png]

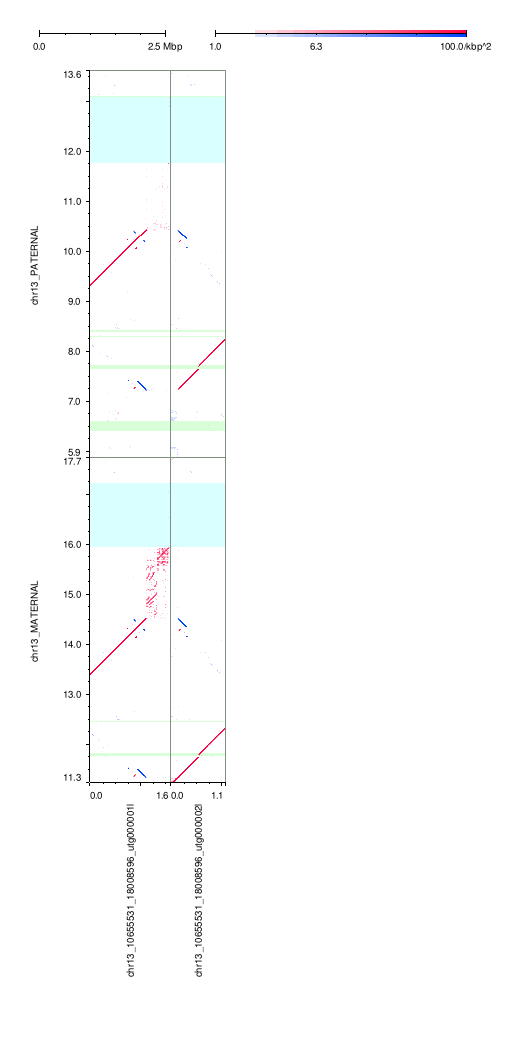

Supplement: btaf648_Supplementary_Data [file btaf648_supplementary_data.zip › Supplementary_Data/supplementary_figures/main_experiment/chr13/mm2-k133-hpc.png]

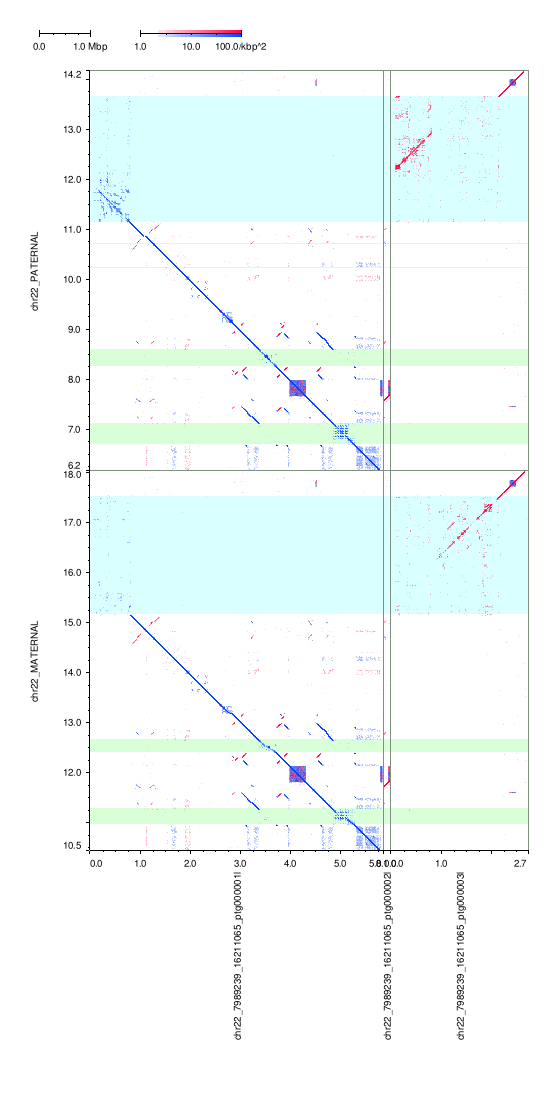

Supplement: btaf648_Supplementary_Data [file btaf648_supplementary_data.zip › Supplementary_Data/supplementary_figures/main_experiment/chr22/hifiasm-ont-r1.png]

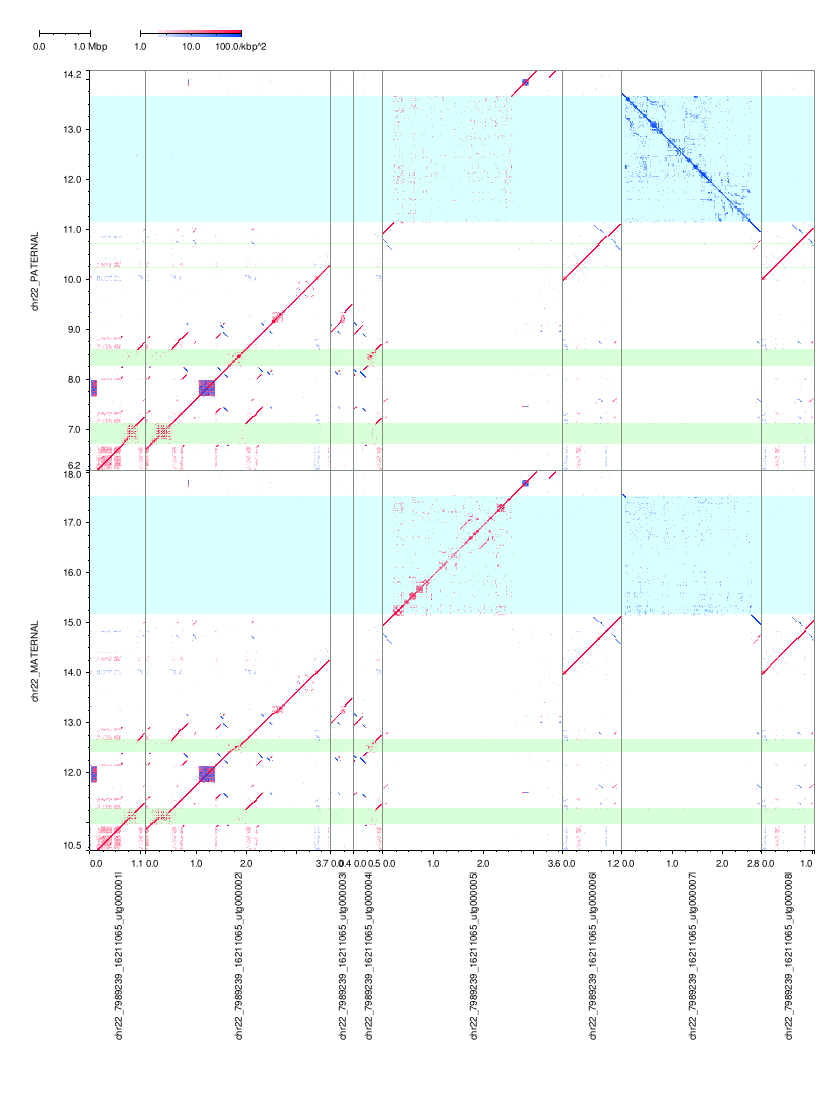

Supplement: btaf648_Supplementary_Data [file btaf648_supplementary_data.zip › Supplementary_Data/supplementary_figures/main_experiment/chr22/mm2-ivh-hpc.png]

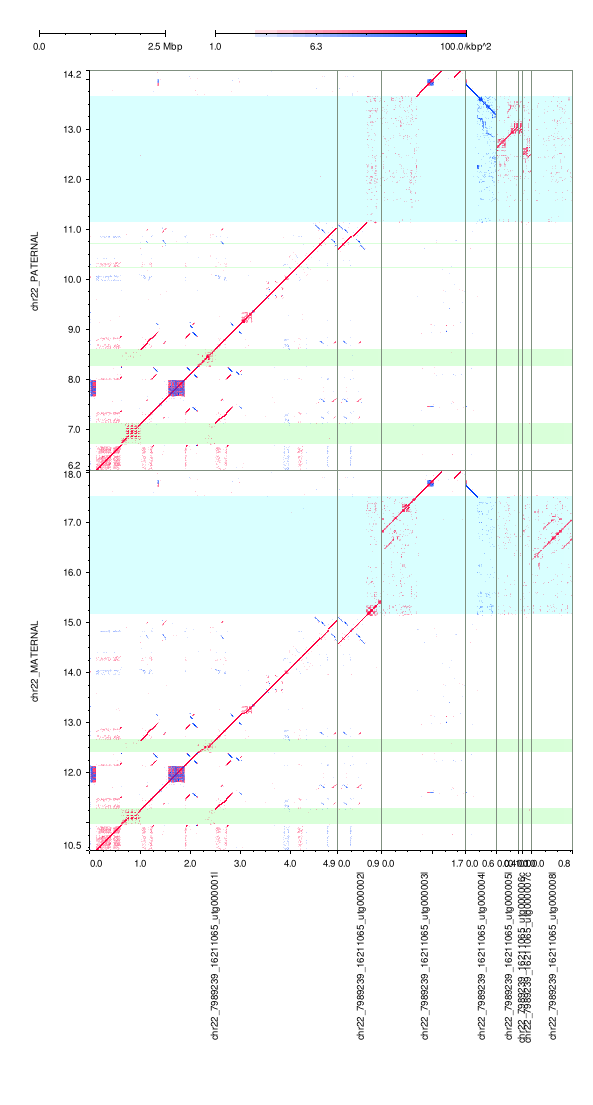

Supplement: btaf648_Supplementary_Data [file btaf648_supplementary_data.zip › Supplementary_Data/supplementary_figures/main_experiment/chr22/mm2-k133-hpc.png]

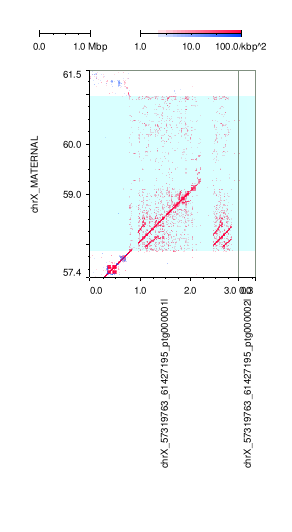

Supplement: btaf648_Supplementary_Data [file btaf648_supplementary_data.zip › Supplementary_Data/supplementary_figures/main_experiment/chrX/hifiasm-ont-r1.png]

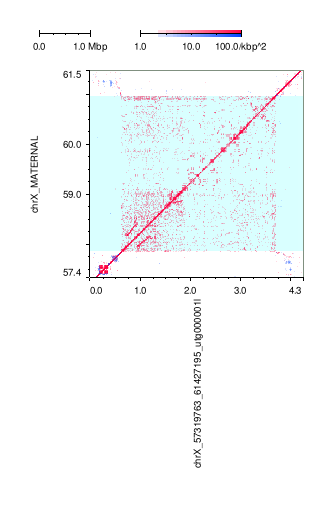

Supplement: btaf648_Supplementary_Data [file btaf648_supplementary_data.zip › Supplementary_Data/supplementary_figures/main_experiment/chrX/mm2-ivh-hpc.png]

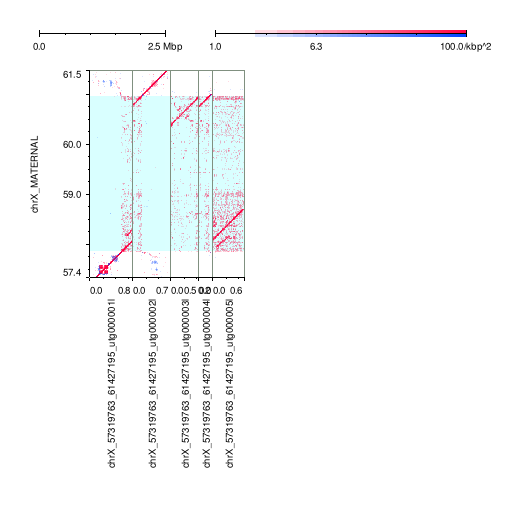

Supplement: btaf648_Supplementary_Data [file btaf648_supplementary_data.zip › Supplementary_Data/supplementary_figures/main_experiment/chrX/mm2-k133-hpc.png]

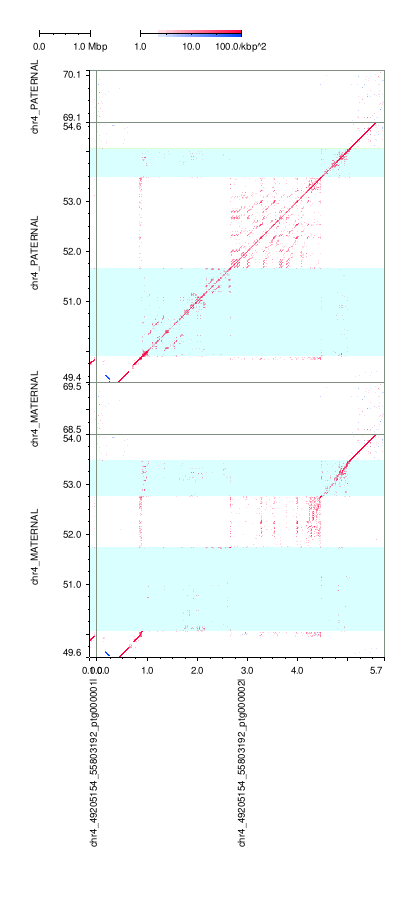

Supplement: btaf648_Supplementary_Data [file btaf648_supplementary_data.zip › Supplementary_Data/supplementary_figures/main_experiment/chr4/hifiasm-ont-r1.png]

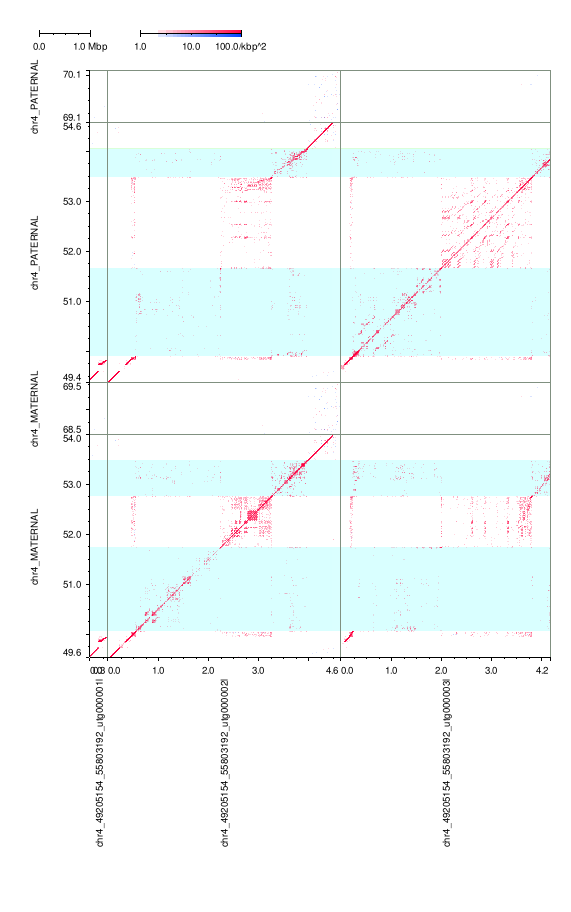

Supplement: btaf648_Supplementary_Data [file btaf648_supplementary_data.zip › Supplementary_Data/supplementary_figures/main_experiment/chr4/mm2-ivh-hpc.png]

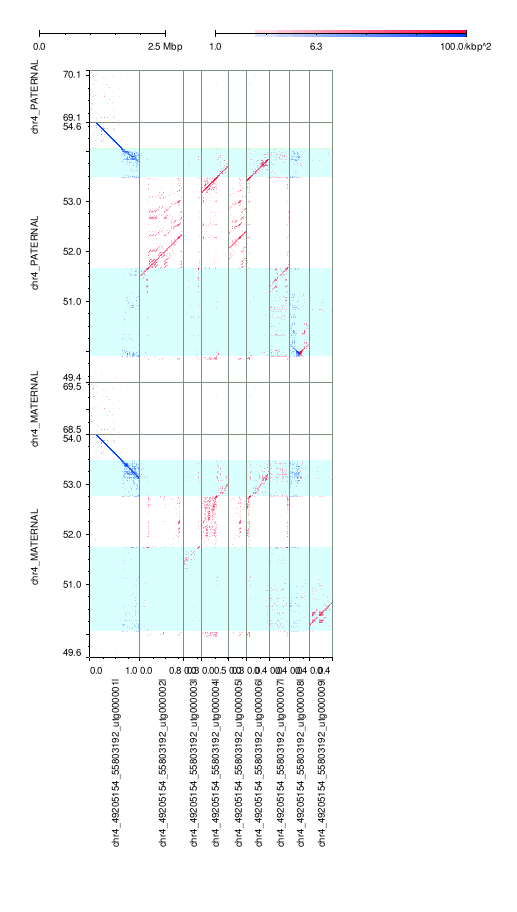

Supplement: btaf648_Supplementary_Data [file btaf648_supplementary_data.zip › Supplementary_Data/supplementary_figures/main_experiment/chr4/mm2-k133-hpc.png]

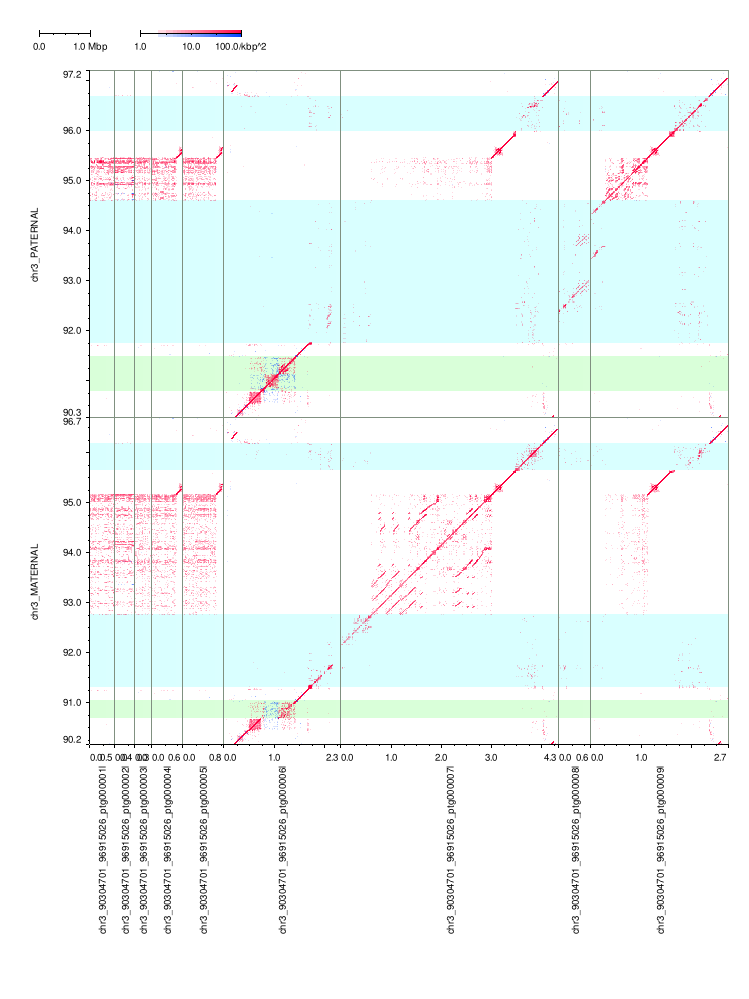

Supplement: btaf648_Supplementary_Data [file btaf648_supplementary_data.zip › Supplementary_Data/supplementary_figures/main_experiment/chr3/hifiasm-ont-r1.png]

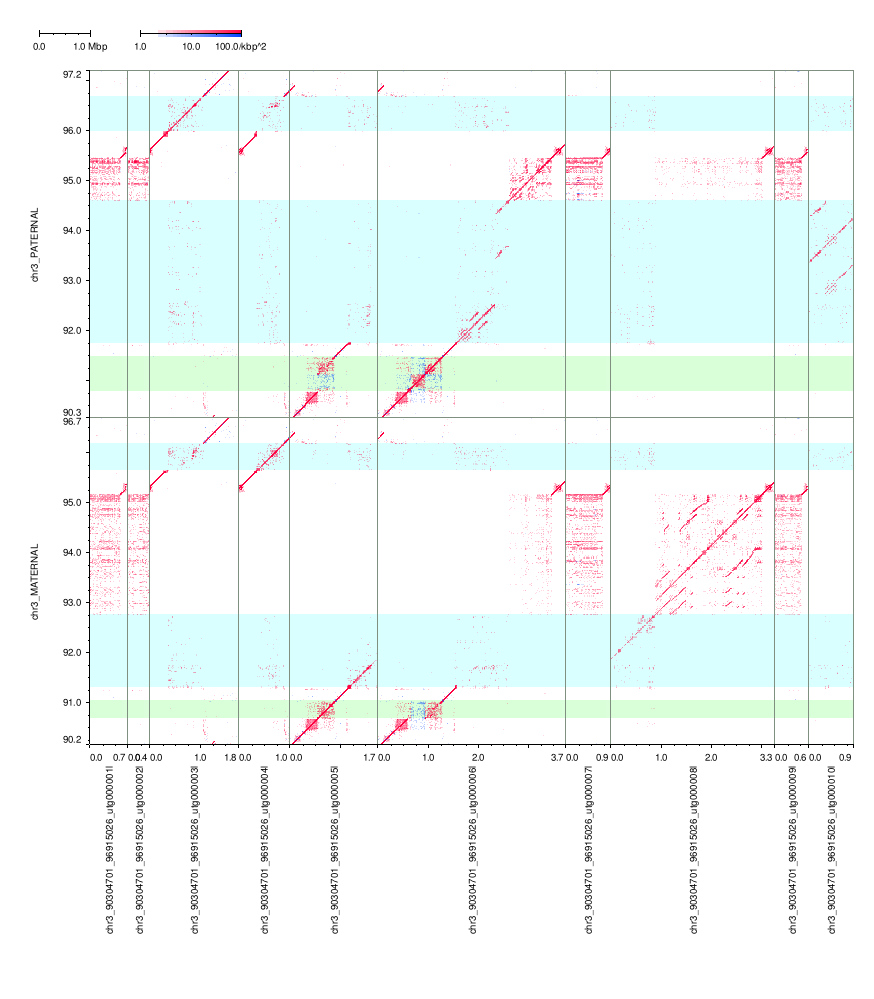

Supplement: btaf648_Supplementary_Data [file btaf648_supplementary_data.zip › Supplementary_Data/supplementary_figures/main_experiment/chr3/mm2-ivh-hpc.png]

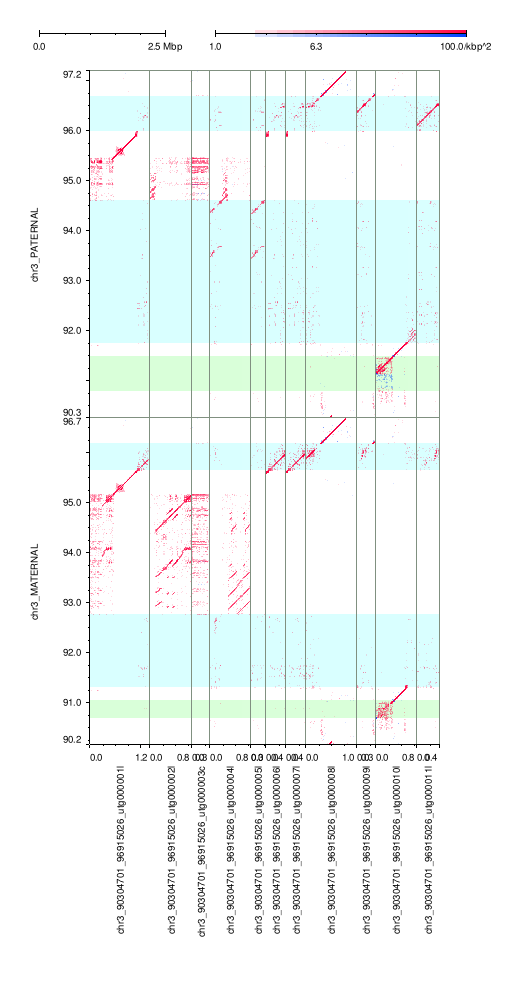

Supplement: btaf648_Supplementary_Data [file btaf648_supplementary_data.zip › Supplementary_Data/supplementary_figures/main_experiment/chr3/mm2-k133-hpc.png]

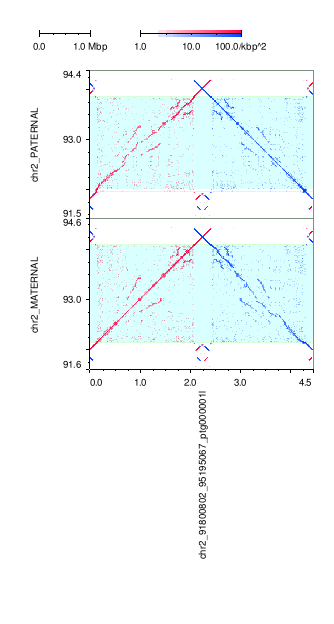

Supplement: btaf648_Supplementary_Data [file btaf648_supplementary_data.zip › Supplementary_Data/supplementary_figures/main_experiment/chr2/hifiasm-ont-r1.png]

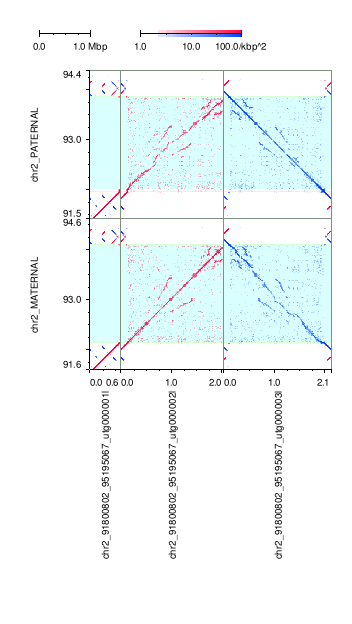

Supplement: btaf648_Supplementary_Data [file btaf648_supplementary_data.zip › Supplementary_Data/supplementary_figures/main_experiment/chr2/mm2-ivh-hpc.png]

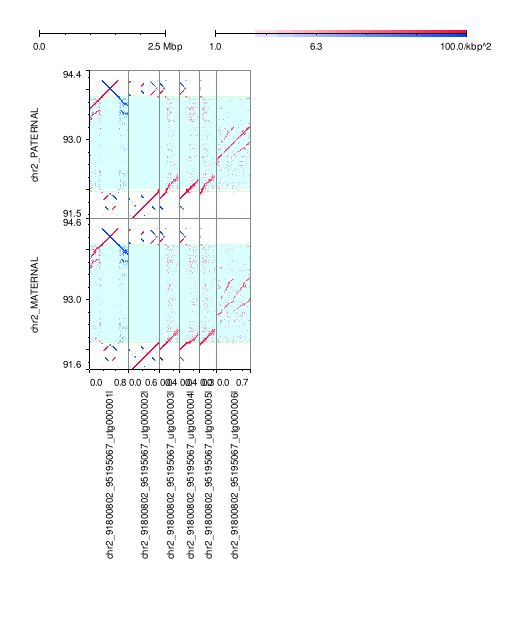

Supplement: btaf648_Supplementary_Data [file btaf648_supplementary_data.zip › Supplementary_Data/supplementary_figures/main_experiment/chr2/mm2-k133-hpc.png]

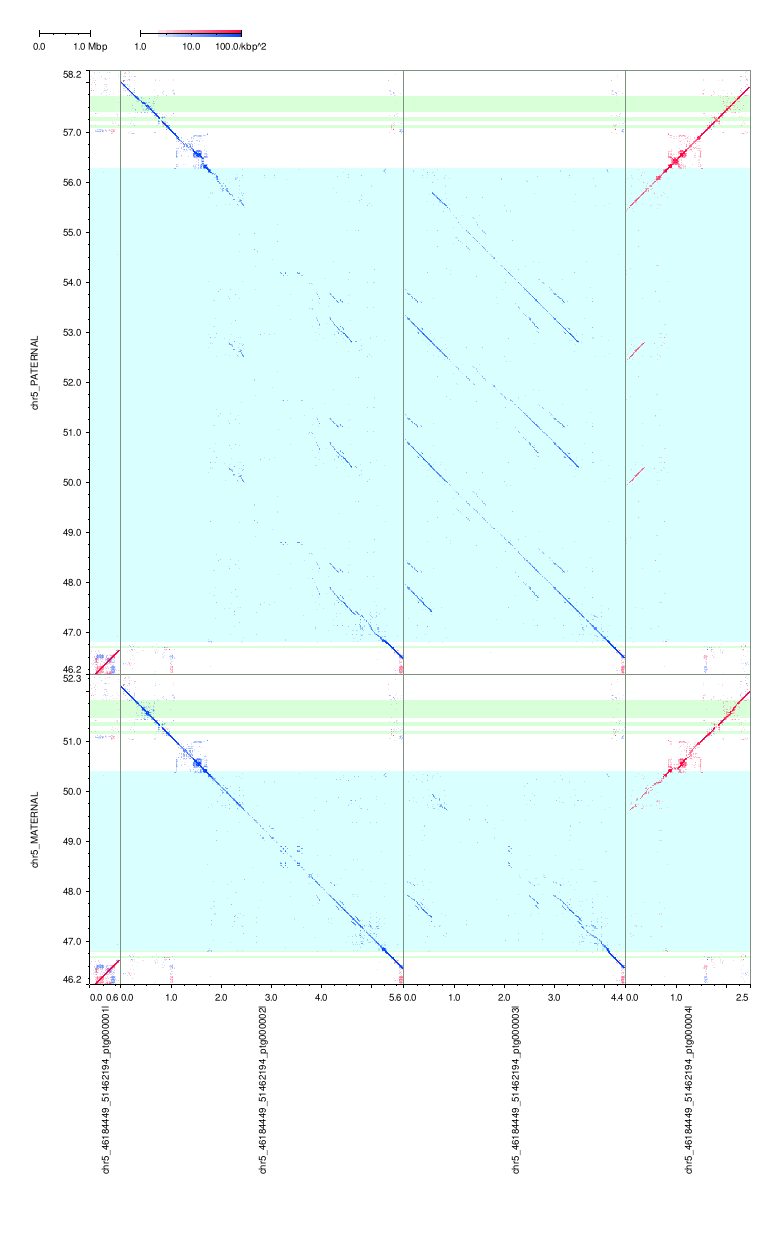

Supplement: btaf648_Supplementary_Data [file btaf648_supplementary_data.zip › Supplementary_Data/supplementary_figures/main_experiment/chr5/hifiasm-ont-r1.png]

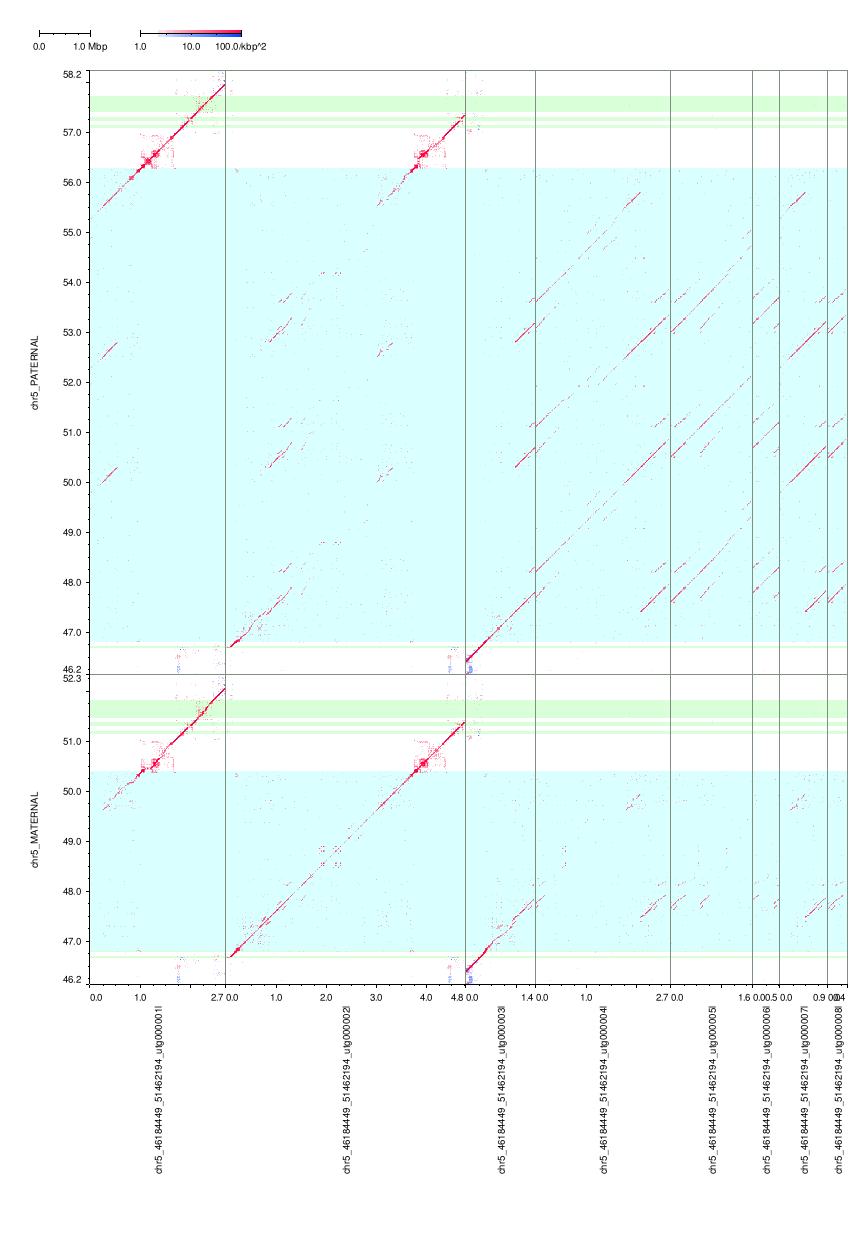

Supplement: btaf648_Supplementary_Data [file btaf648_supplementary_data.zip › Supplementary_Data/supplementary_figures/main_experiment/chr5/mm2-ivh-hpc.png]

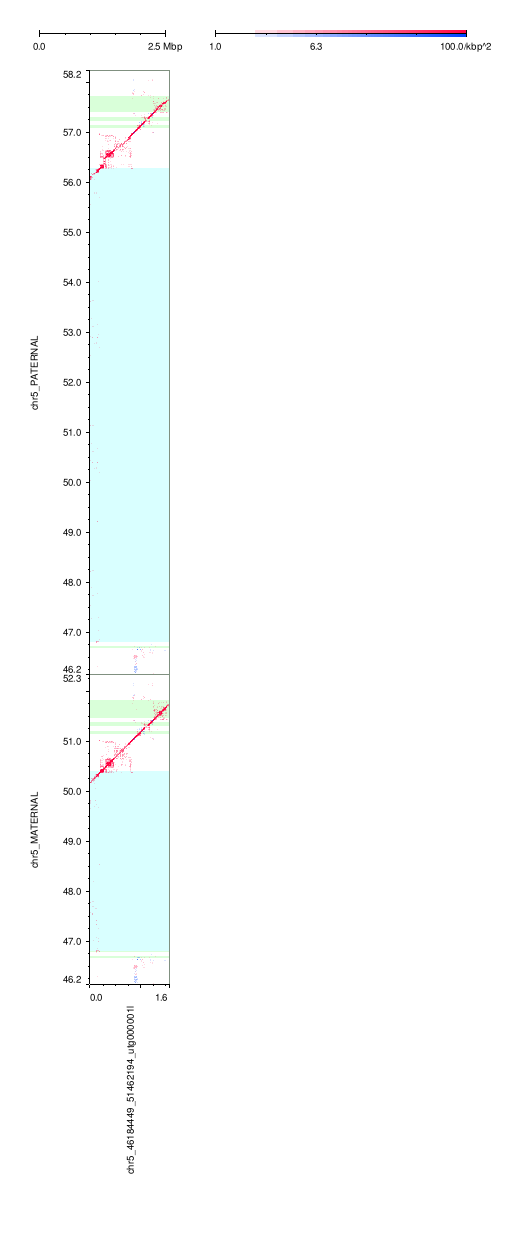

Supplement: btaf648_Supplementary_Data [file btaf648_supplementary_data.zip › Supplementary_Data/supplementary_figures/main_experiment/chr5/mm2-k133-hpc.png]

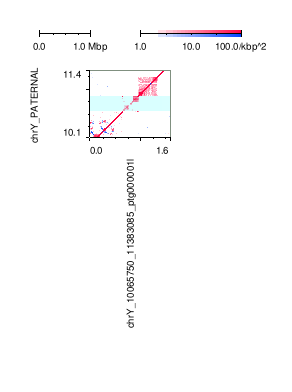

Supplement: btaf648_Supplementary_Data [file btaf648_supplementary_data.zip › Supplementary_Data/supplementary_figures/main_experiment/chrY/hifiasm-ont-r1.png]

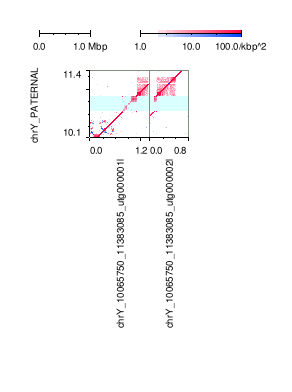

Supplement: btaf648_Supplementary_Data [file btaf648_supplementary_data.zip › Supplementary_Data/supplementary_figures/main_experiment/chrY/mm2-ivh-hpc.png]

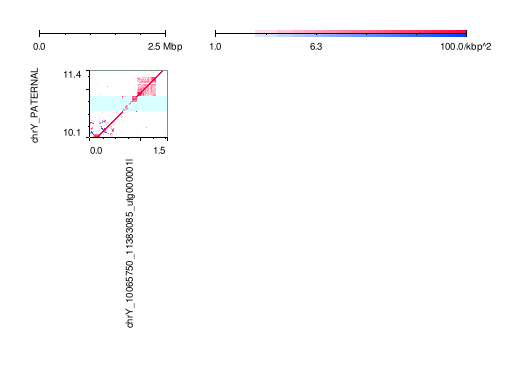

Supplement: btaf648_Supplementary_Data [file btaf648_supplementary_data.zip › Supplementary_Data/supplementary_figures/main_experiment/chrY/mm2-k133-hpc.png]

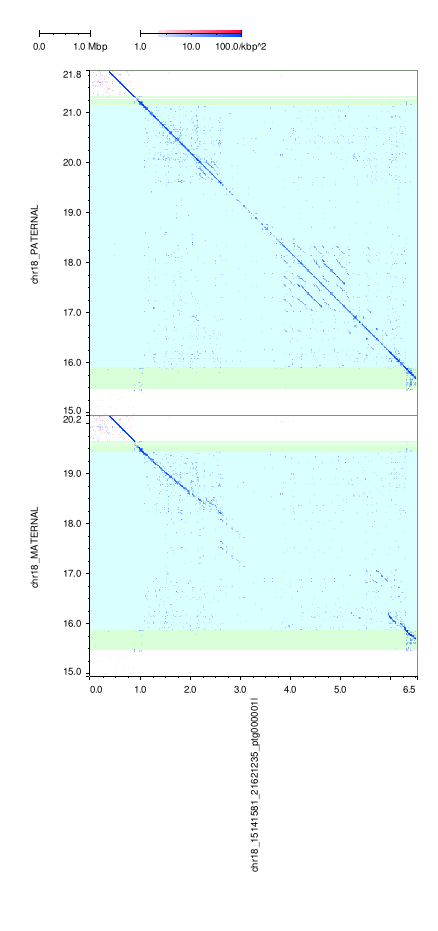

Supplement: btaf648_Supplementary_Data [file btaf648_supplementary_data.zip › Supplementary_Data/supplementary_figures/main_experiment/chr18/hifiasm-ont-r1.png]

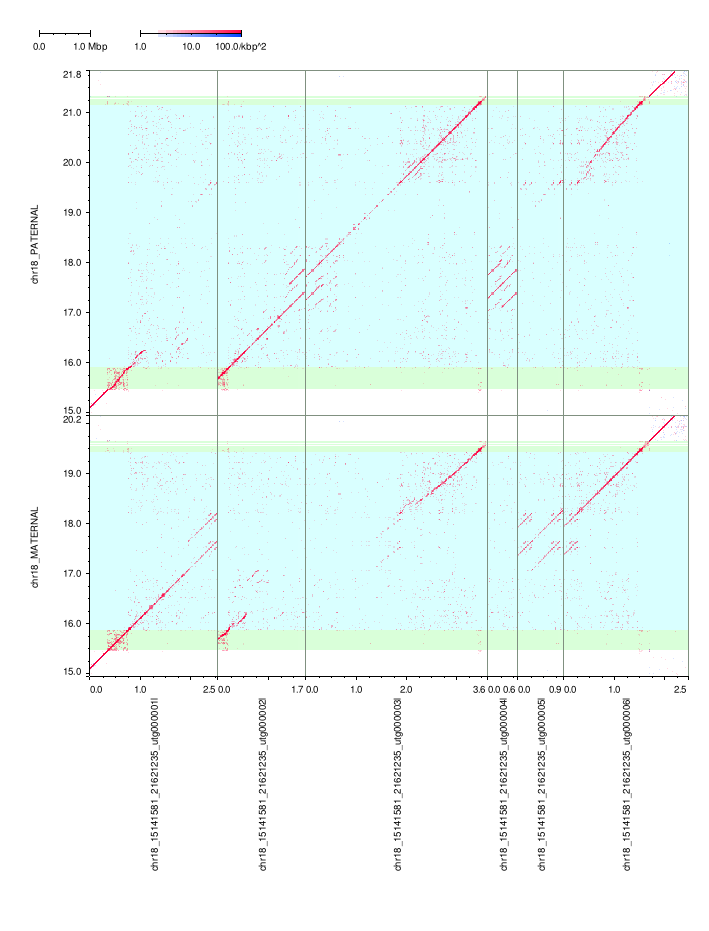

Supplement: btaf648_Supplementary_Data [file btaf648_supplementary_data.zip › Supplementary_Data/supplementary_figures/main_experiment/chr18/mm2-ivh-hpc.png]

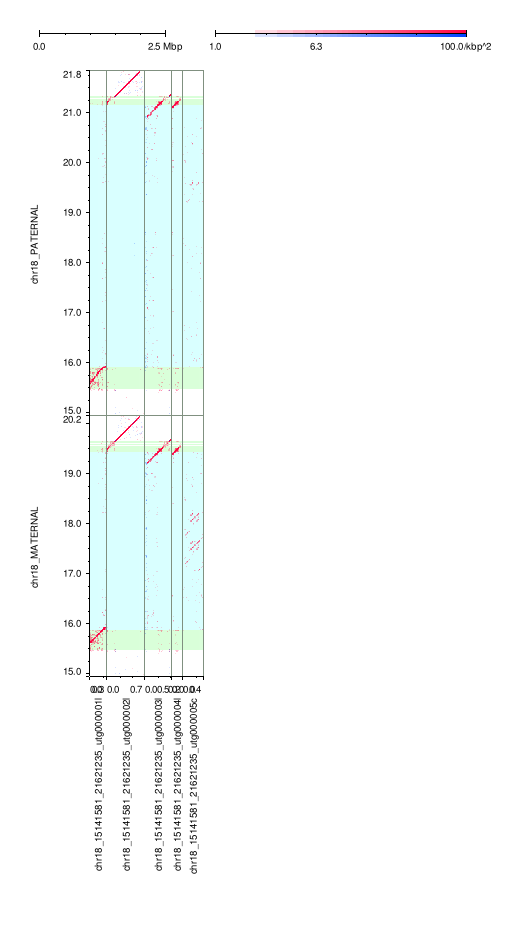

Supplement: btaf648_Supplementary_Data [file btaf648_supplementary_data.zip › Supplementary_Data/supplementary_figures/main_experiment/chr18/mm2-k133-hpc.png]

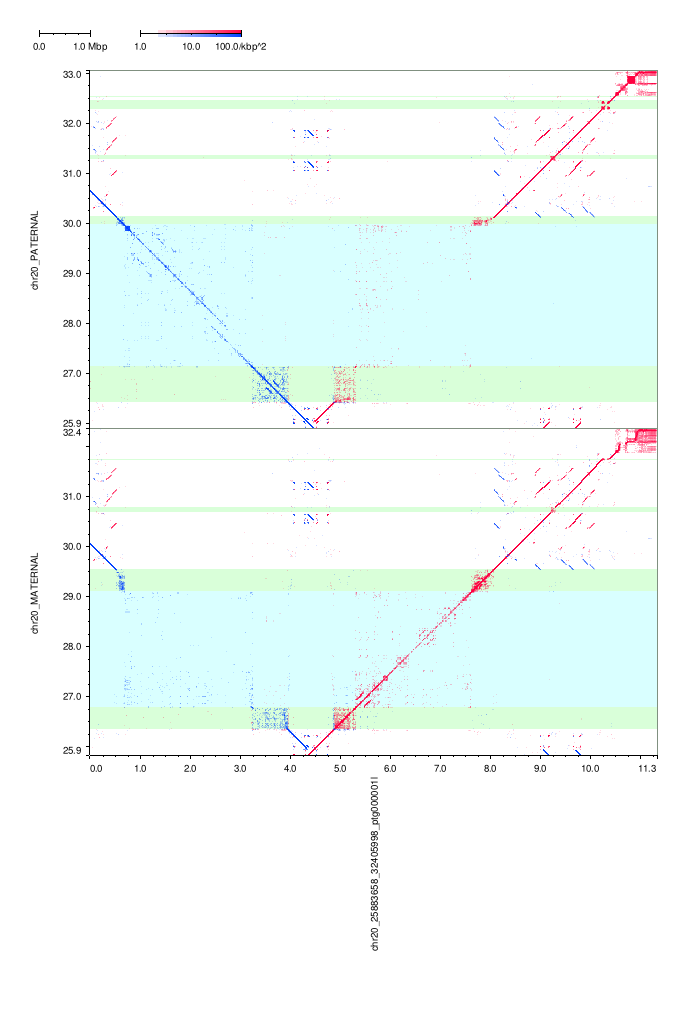

Supplement: btaf648_Supplementary_Data [file btaf648_supplementary_data.zip › Supplementary_Data/supplementary_figures/main_experiment/chr20/hifiasm-ont-r1.png]

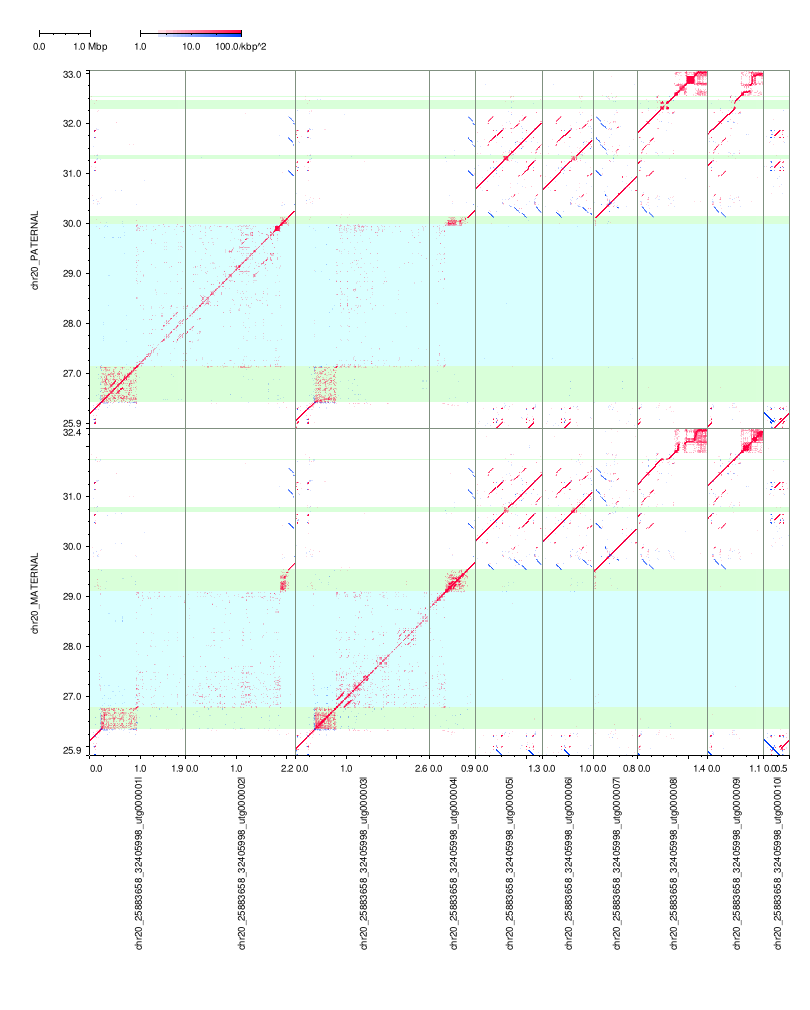

Supplement: btaf648_Supplementary_Data [file btaf648_supplementary_data.zip › Supplementary_Data/supplementary_figures/main_experiment/chr20/mm2-ivh-hpc.png]

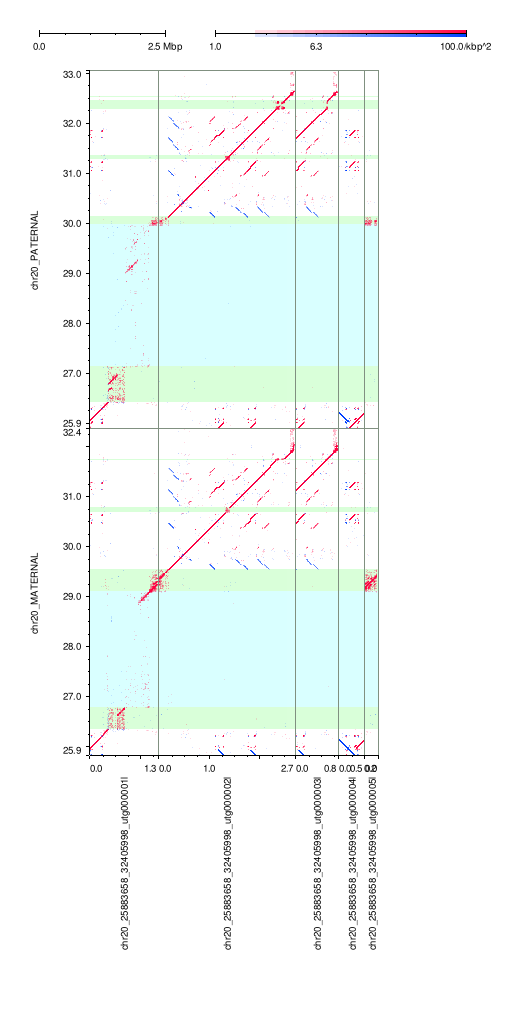

Supplement: btaf648_Supplementary_Data [file btaf648_supplementary_data.zip › Supplementary_Data/supplementary_figures/main_experiment/chr20/mm2-k133-hpc.png]

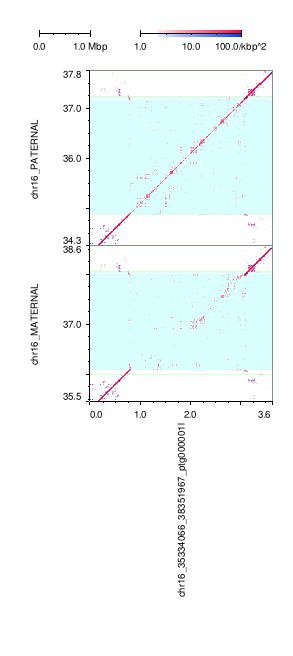

Supplement: btaf648_Supplementary_Data [file btaf648_supplementary_data.zip › Supplementary_Data/supplementary_figures/main_experiment/chr16/hifiasm-ont-r1.png]

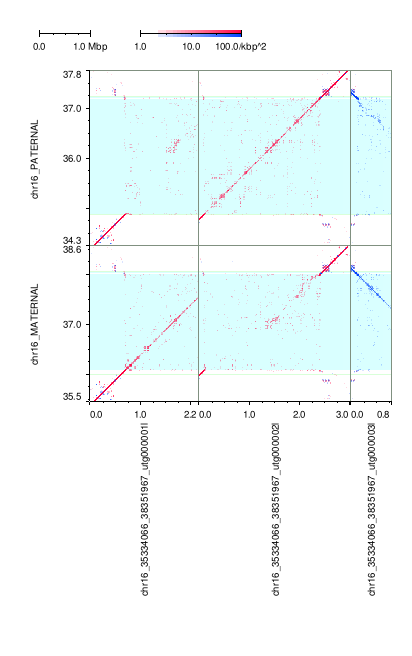

Supplement: btaf648_Supplementary_Data [file btaf648_supplementary_data.zip › Supplementary_Data/supplementary_figures/main_experiment/chr16/mm2-ivh-hpc.png]

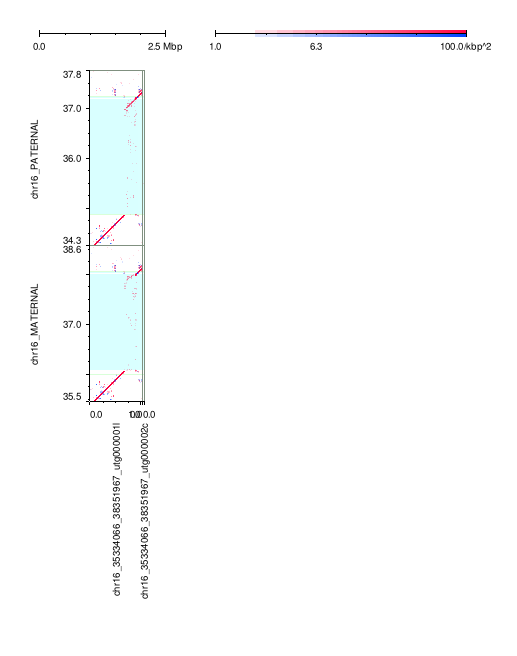

Supplement: btaf648_Supplementary_Data [file btaf648_supplementary_data.zip › Supplementary_Data/supplementary_figures/main_experiment/chr16/mm2-k133-hpc.png]

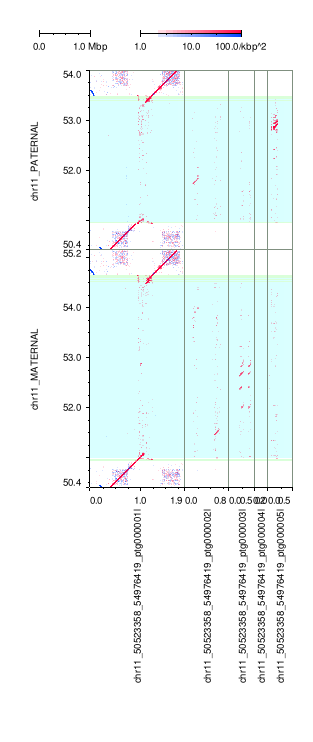

Supplement: btaf648_Supplementary_Data [file btaf648_supplementary_data.zip › Supplementary_Data/supplementary_figures/main_experiment/chr11/hifiasm-ont-r1.png]

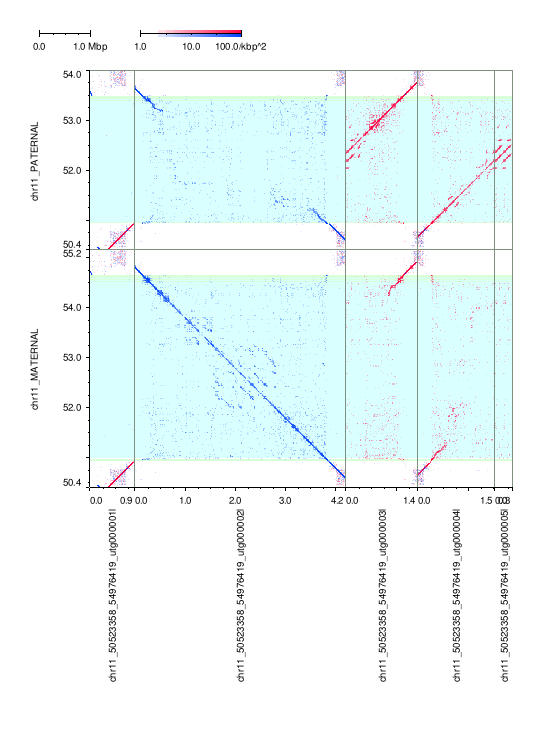

Supplement: btaf648_Supplementary_Data [file btaf648_supplementary_data.zip › Supplementary_Data/supplementary_figures/main_experiment/chr11/mm2-ivh-hpc.png]

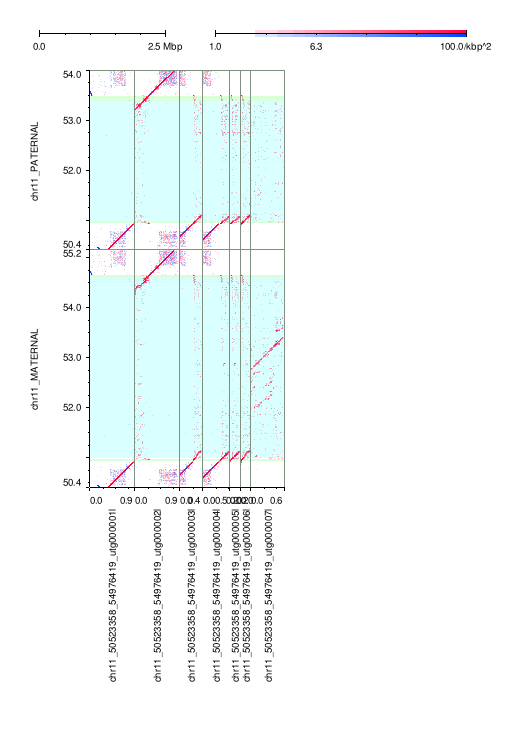

Supplement: btaf648_Supplementary_Data [file btaf648_supplementary_data.zip › Supplementary_Data/supplementary_figures/main_experiment/chr11/mm2-k133-hpc.png]

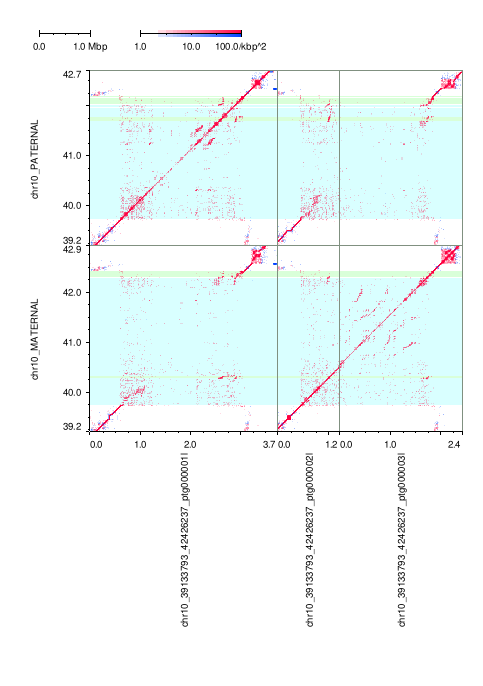

Supplement: btaf648_Supplementary_Data [file btaf648_supplementary_data.zip › Supplementary_Data/supplementary_figures/main_experiment/chr10/hifiasm-ont-r1.png]

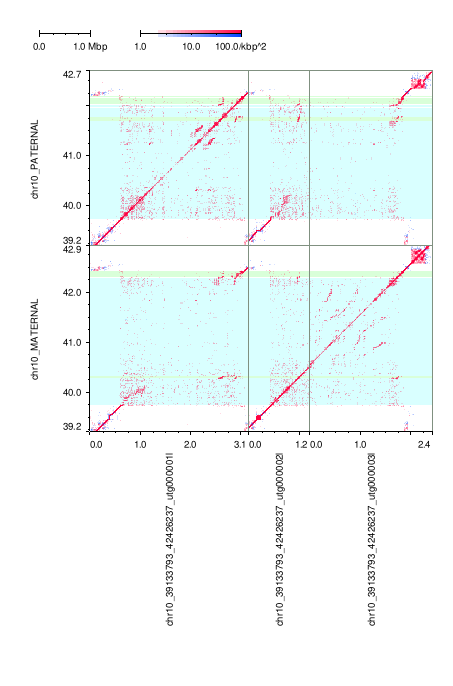

Supplement: btaf648_Supplementary_Data [file btaf648_supplementary_data.zip › Supplementary_Data/supplementary_figures/main_experiment/chr10/mm2-ivh-hpc.png]

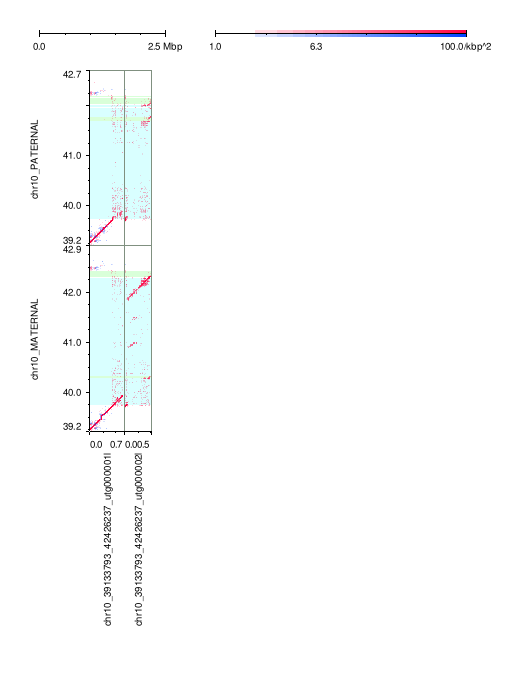

Supplement: btaf648_Supplementary_Data [file btaf648_supplementary_data.zip › Supplementary_Data/supplementary_figures/main_experiment/chr10/mm2-k133-hpc.png]

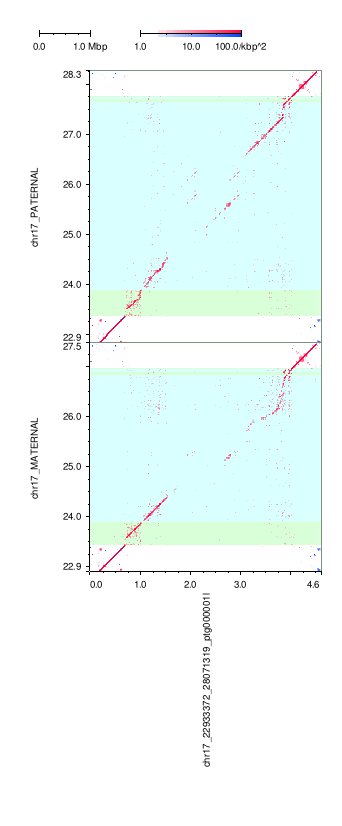

Supplement: btaf648_Supplementary_Data [file btaf648_supplementary_data.zip › Supplementary_Data/supplementary_figures/main_experiment/chr17/hifiasm-ont-r1.png]

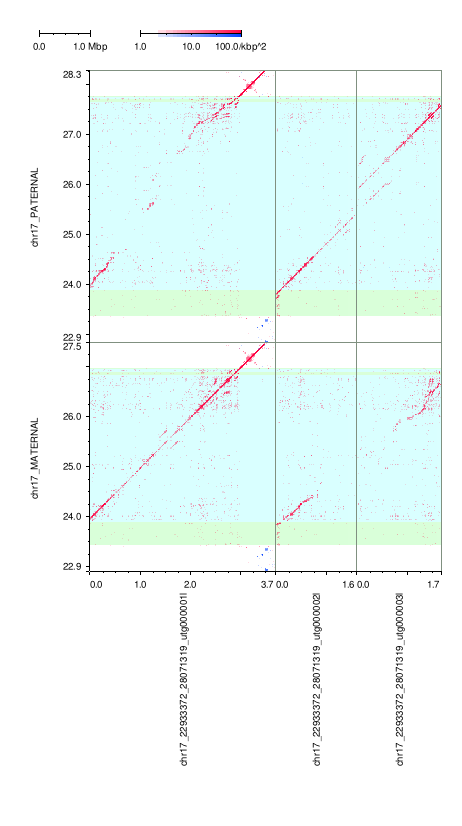

Supplement: btaf648_Supplementary_Data [file btaf648_supplementary_data.zip › Supplementary_Data/supplementary_figures/main_experiment/chr17/mm2-ivh-hpc.png]

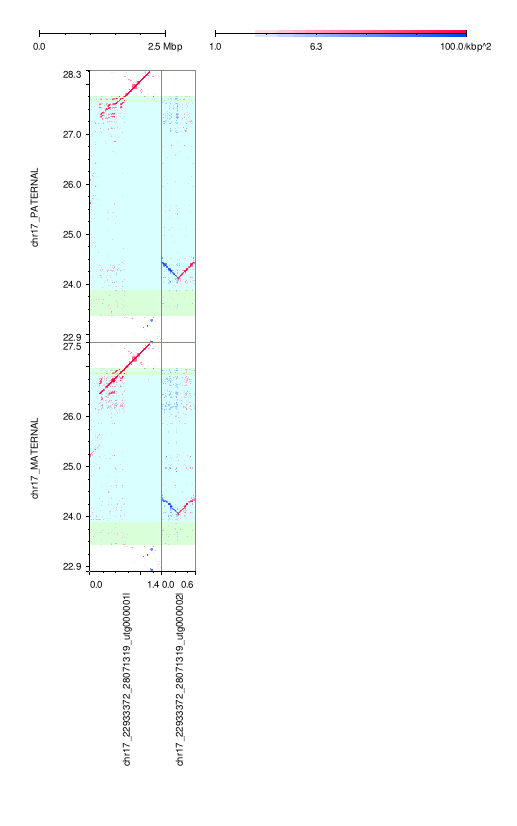

Supplement: btaf648_Supplementary_Data [file btaf648_supplementary_data.zip › Supplementary_Data/supplementary_figures/main_experiment/chr17/mm2-k133-hpc.png]

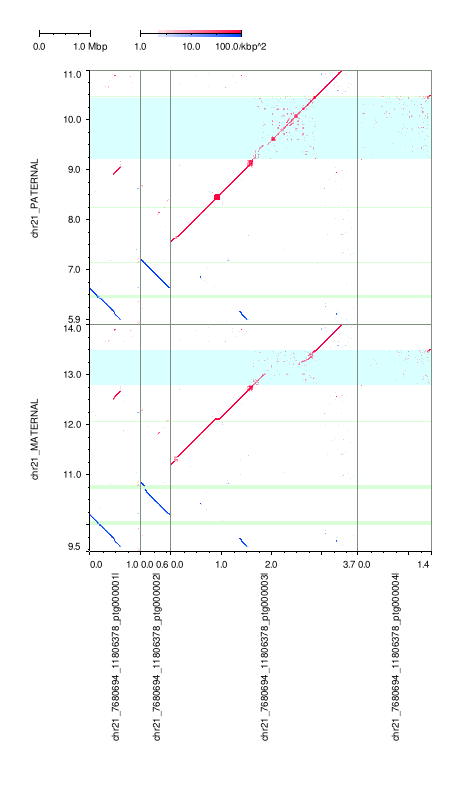

Supplement: btaf648_Supplementary_Data [file btaf648_supplementary_data.zip › Supplementary_Data/supplementary_figures/main_experiment/chr21/hifiasm-ont-r1.png]

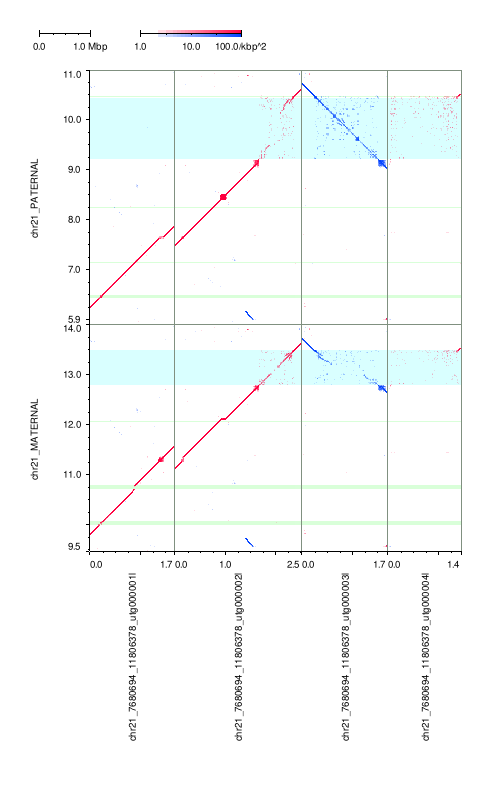

Supplement: btaf648_Supplementary_Data [file btaf648_supplementary_data.zip › Supplementary_Data/supplementary_figures/main_experiment/chr21/mm2-ivh-hpc.png]

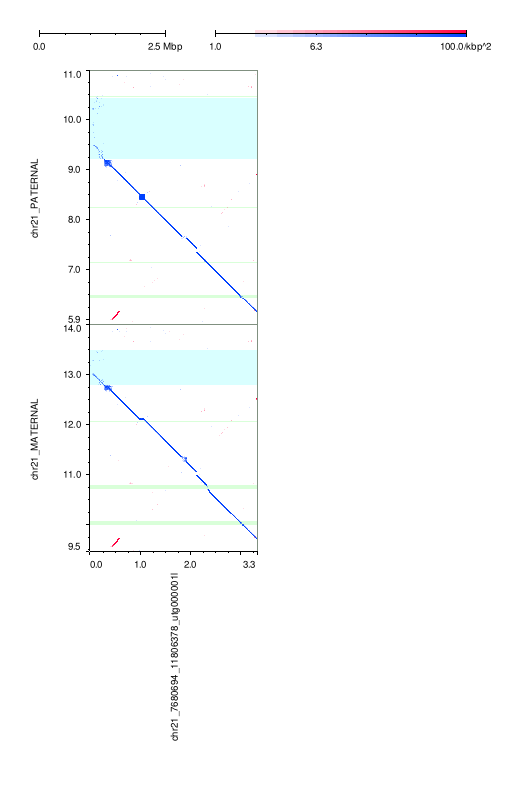

Supplement: btaf648_Supplementary_Data [file btaf648_supplementary_data.zip › Supplementary_Data/supplementary_figures/main_experiment/chr21/mm2-k133-hpc.png]

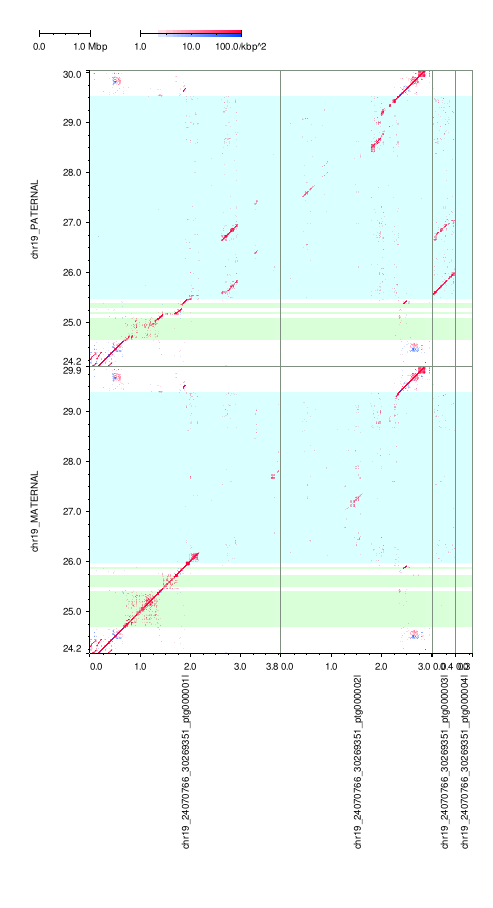

Supplement: btaf648_Supplementary_Data [file btaf648_supplementary_data.zip › Supplementary_Data/supplementary_figures/main_experiment/chr19/hifiasm-ont-r1.png]

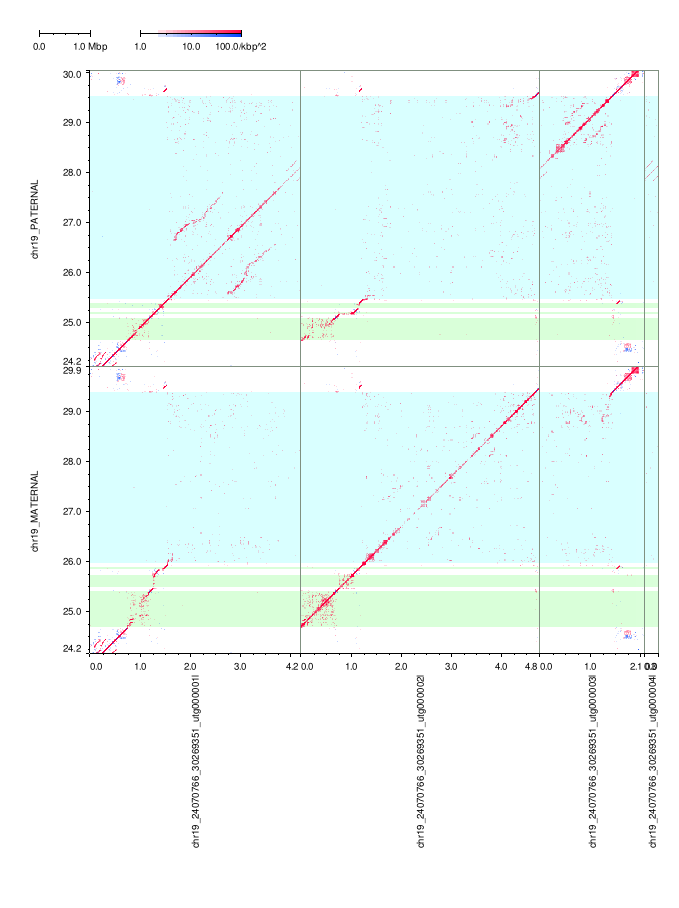

Supplement: btaf648_Supplementary_Data [file btaf648_supplementary_data.zip › Supplementary_Data/supplementary_figures/main_experiment/chr19/mm2-ivh-hpc.png]

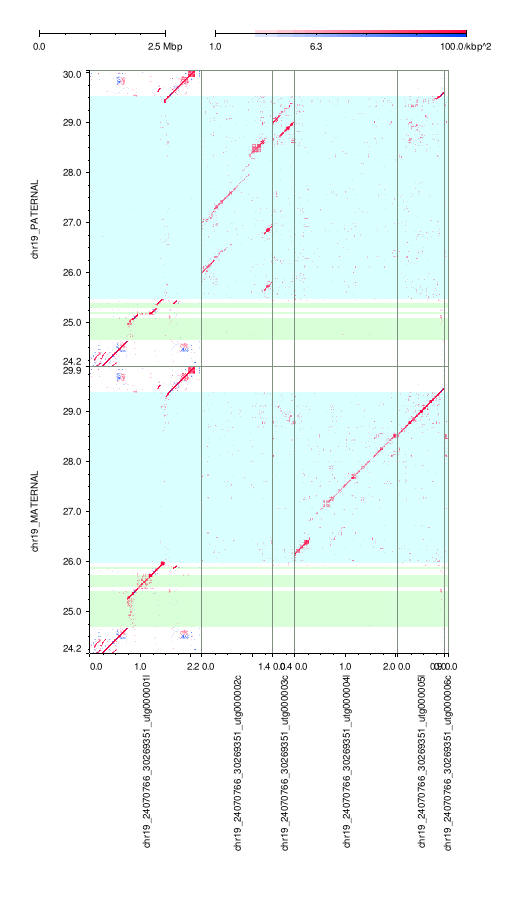

Supplement: btaf648_Supplementary_Data [file btaf648_supplementary_data.zip › Supplementary_Data/supplementary_figures/main_experiment/chr19/mm2-k133-hpc.png]

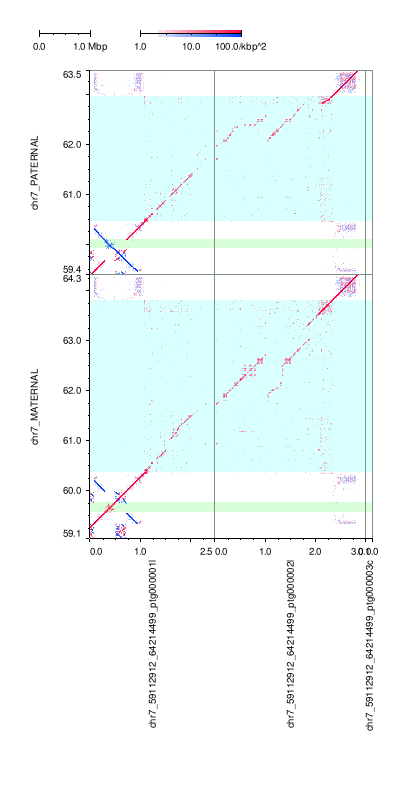

Supplement: btaf648_Supplementary_Data [file btaf648_supplementary_data.zip › Supplementary_Data/supplementary_figures/main_experiment/chr7/hifiasm-ont-r1.png]

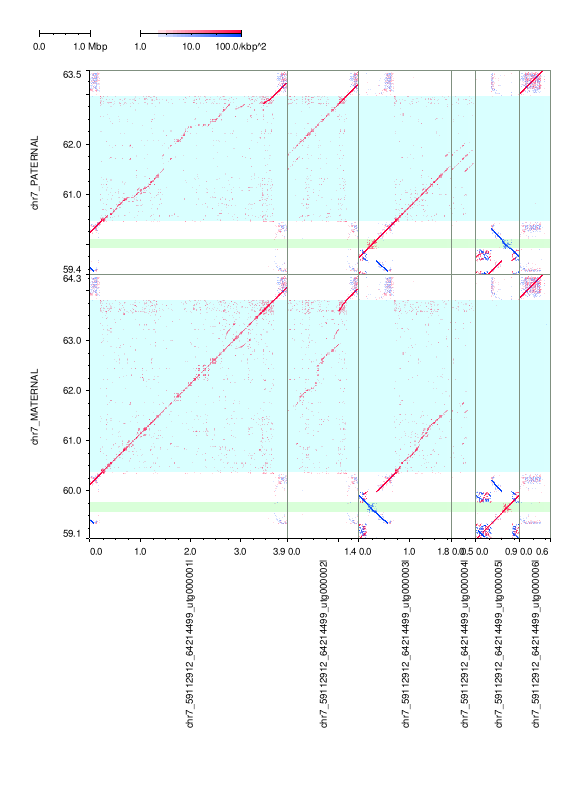

Supplement: btaf648_Supplementary_Data [file btaf648_supplementary_data.zip › Supplementary_Data/supplementary_figures/main_experiment/chr7/mm2-ivh-hpc.png]

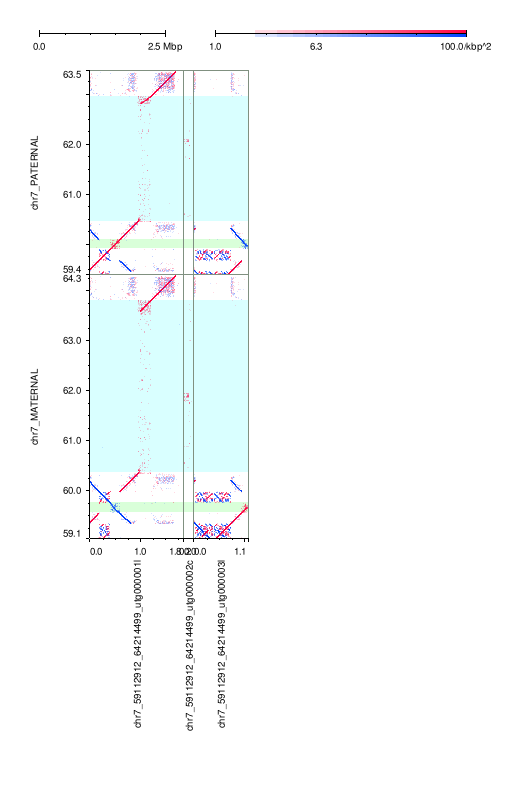

Supplement: btaf648_Supplementary_Data [file btaf648_supplementary_data.zip › Supplementary_Data/supplementary_figures/main_experiment/chr7/mm2-k133-hpc.png]

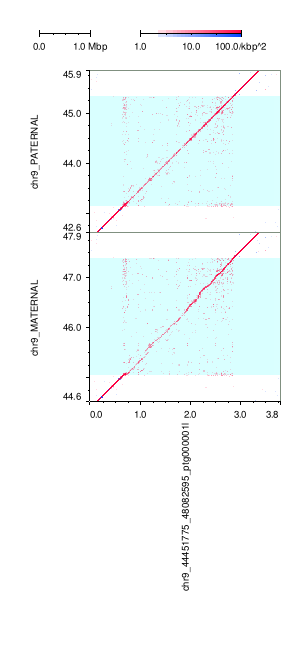

Supplement: btaf648_Supplementary_Data [file btaf648_supplementary_data.zip › Supplementary_Data/supplementary_figures/main_experiment/chr9/hifiasm-ont-r1.png]

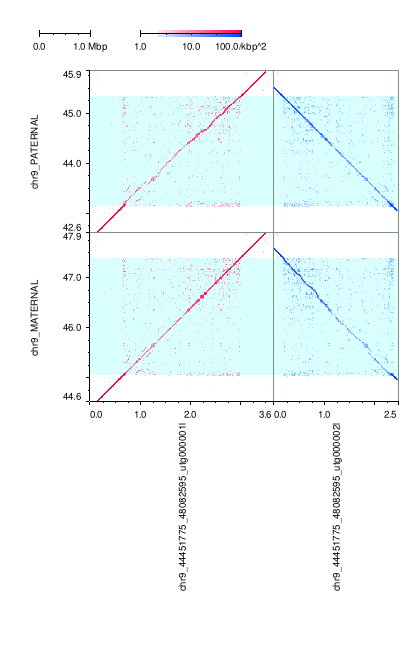

Supplement: btaf648_Supplementary_Data [file btaf648_supplementary_data.zip › Supplementary_Data/supplementary_figures/main_experiment/chr9/mm2-ivh-hpc.png]

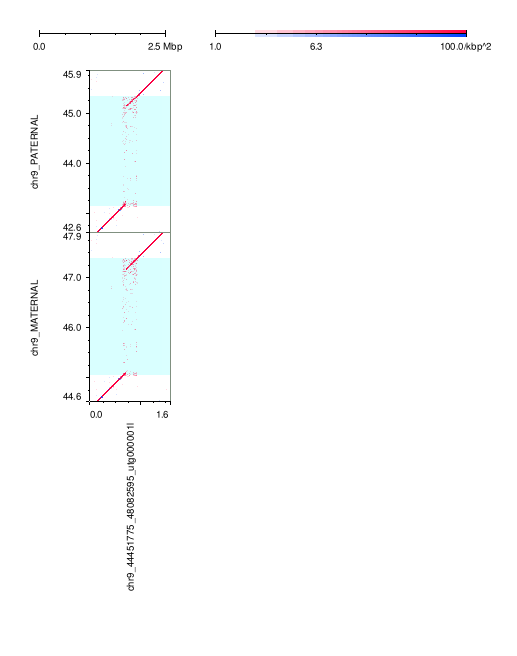

Supplement: btaf648_Supplementary_Data [file btaf648_supplementary_data.zip › Supplementary_Data/supplementary_figures/main_experiment/chr9/mm2-k133-hpc.png]

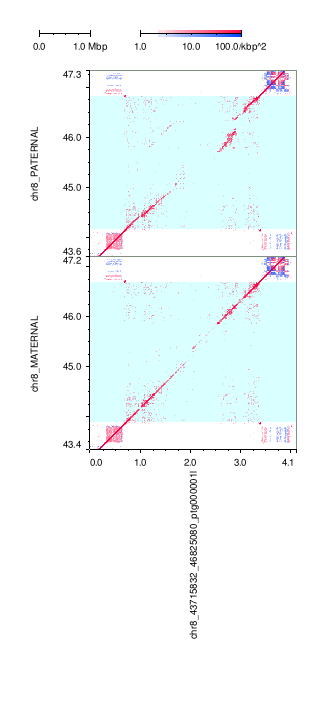

Supplement: btaf648_Supplementary_Data [file btaf648_supplementary_data.zip › Supplementary_Data/supplementary_figures/main_experiment/chr8/hifiasm-ont-r1.png]

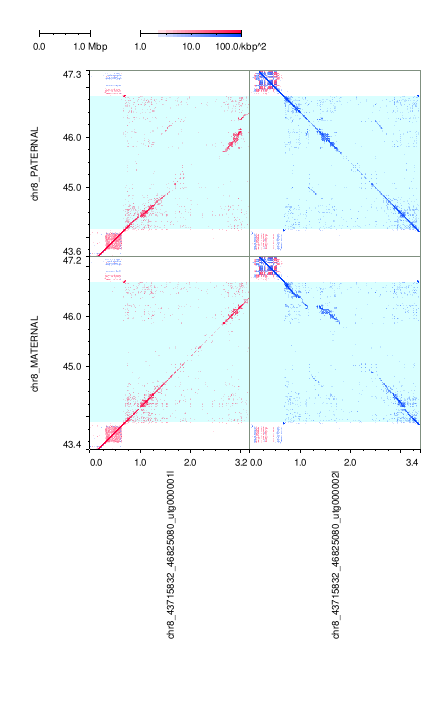

Supplement: btaf648_Supplementary_Data [file btaf648_supplementary_data.zip › Supplementary_Data/supplementary_figures/main_experiment/chr8/mm2-ivh-hpc.png]

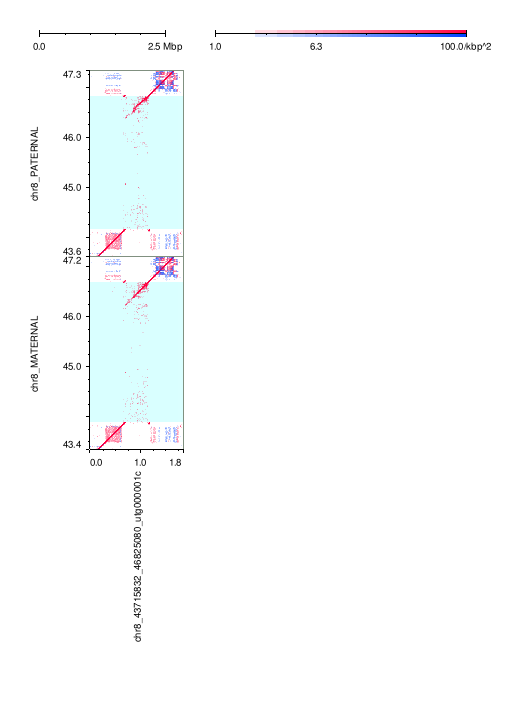

Supplement: btaf648_Supplementary_Data [file btaf648_supplementary_data.zip › Supplementary_Data/supplementary_figures/main_experiment/chr8/mm2-k133-hpc.png]

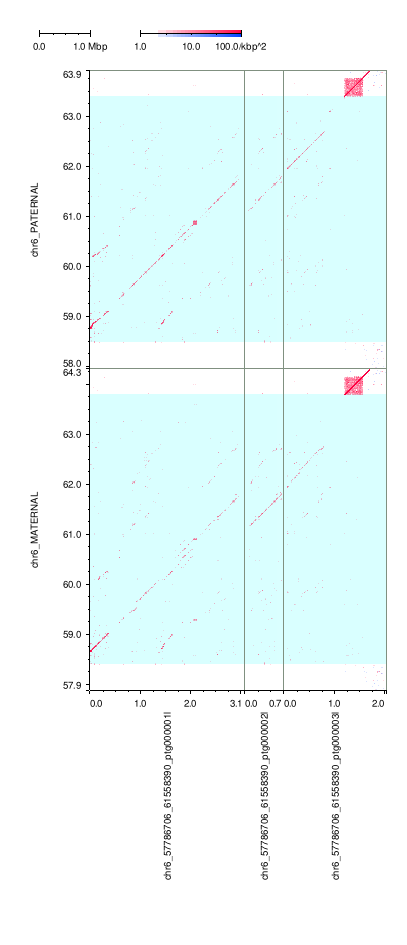

Supplement: btaf648_Supplementary_Data [file btaf648_supplementary_data.zip › Supplementary_Data/supplementary_figures/main_experiment/chr6/hifiasm-ont-r1.png]

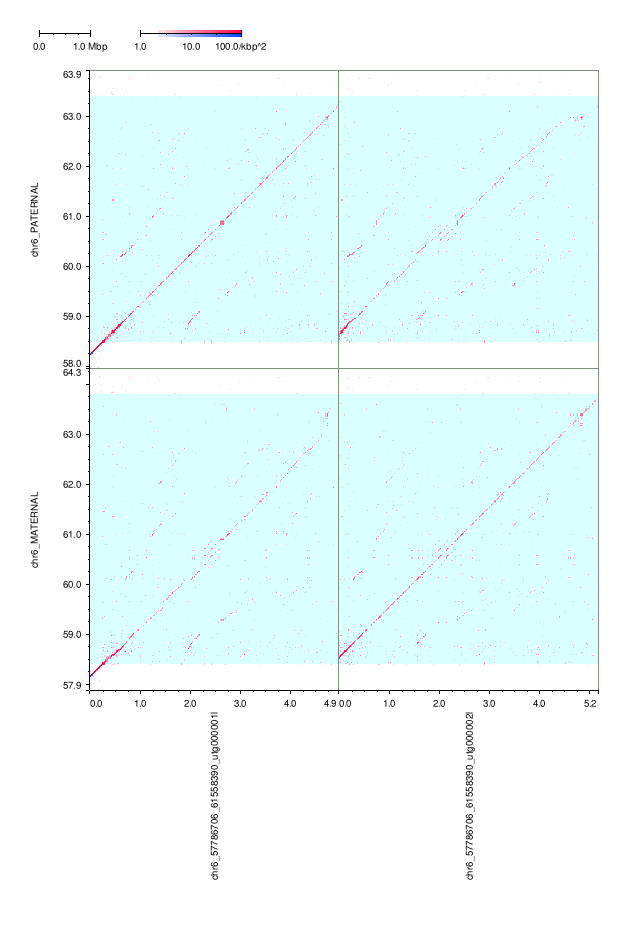

Supplement: btaf648_Supplementary_Data [file btaf648_supplementary_data.zip › Supplementary_Data/supplementary_figures/main_experiment/chr6/mm2-ivh-hpc.png]

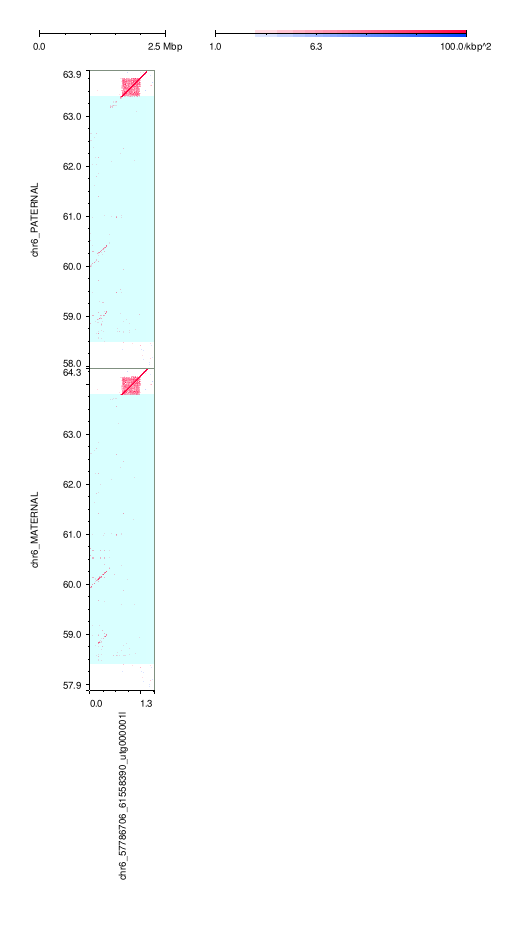

Supplement: btaf648_Supplementary_Data [file btaf648_supplementary_data.zip › Supplementary_Data/supplementary_figures/main_experiment/chr6/mm2-k133-hpc.png]

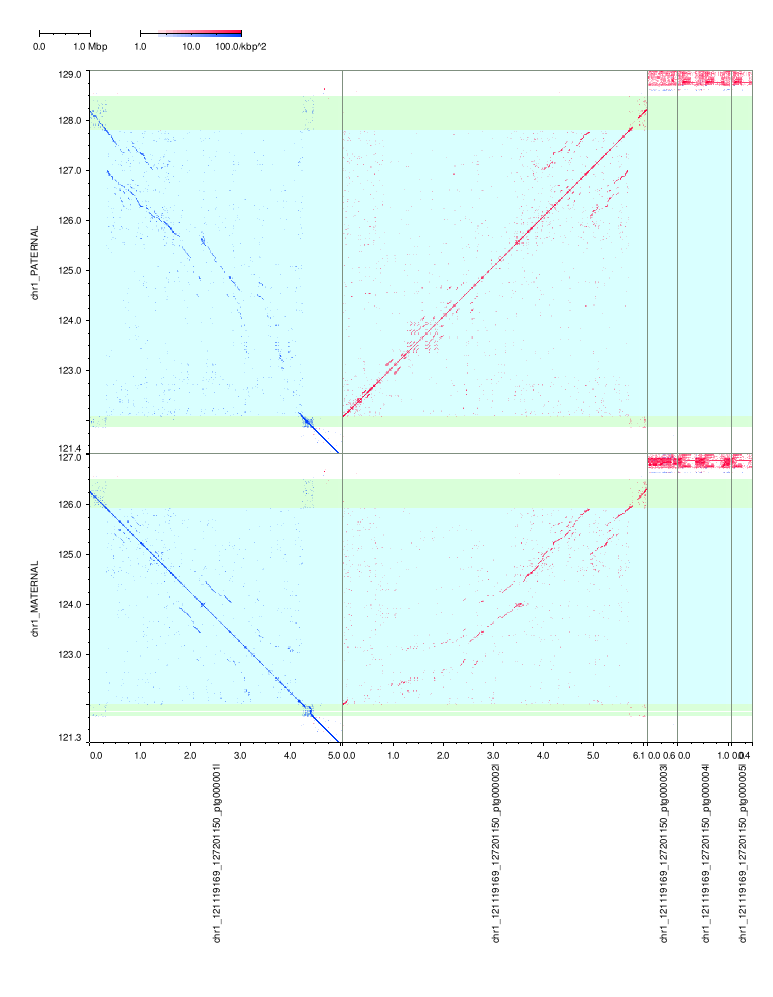

Supplement: btaf648_Supplementary_Data [file btaf648_supplementary_data.zip › Supplementary_Data/supplementary_figures/main_experiment/chr1/hifiasm-ont-r1.png]

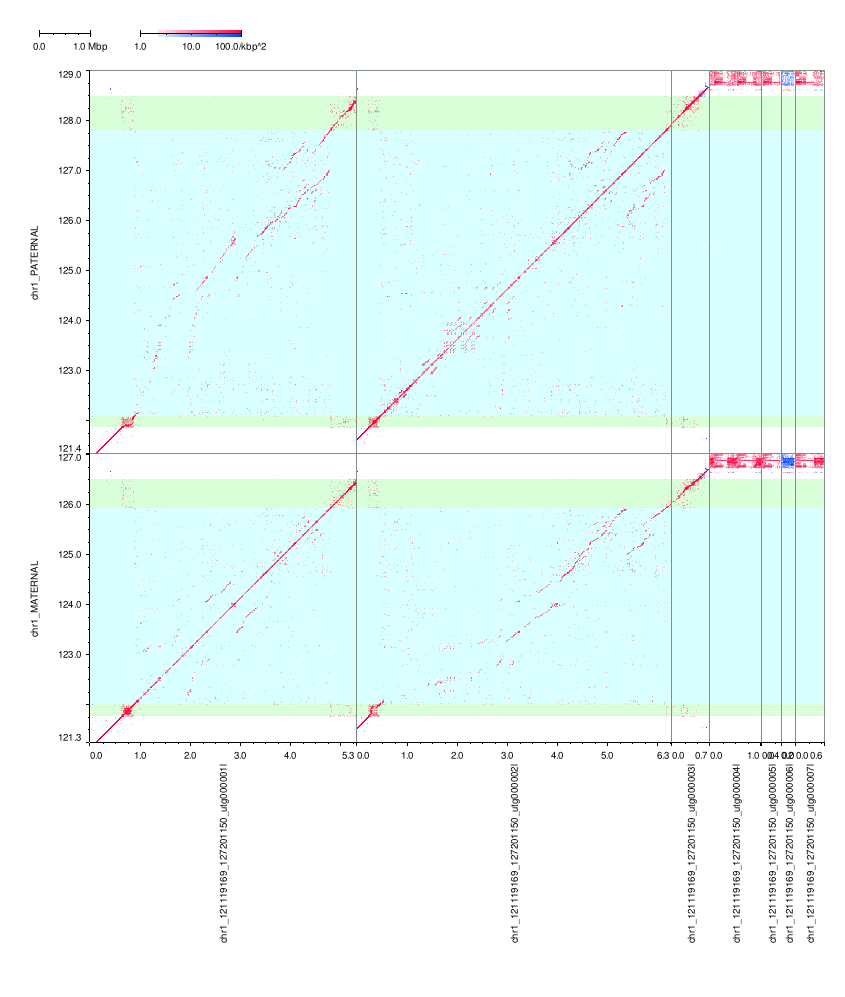

Supplement: btaf648_Supplementary_Data [file btaf648_supplementary_data.zip › Supplementary_Data/supplementary_figures/main_experiment/chr1/mm2-ivh-hpc.png]

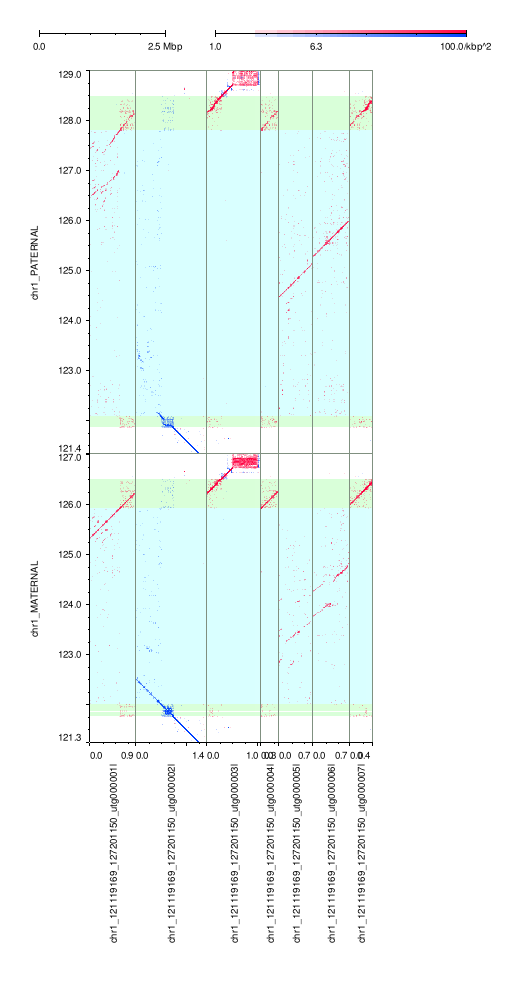

Supplement: btaf648_Supplementary_Data [file btaf648_supplementary_data.zip › Supplementary_Data/supplementary_figures/main_experiment/chr1/mm2-k133-hpc.png]

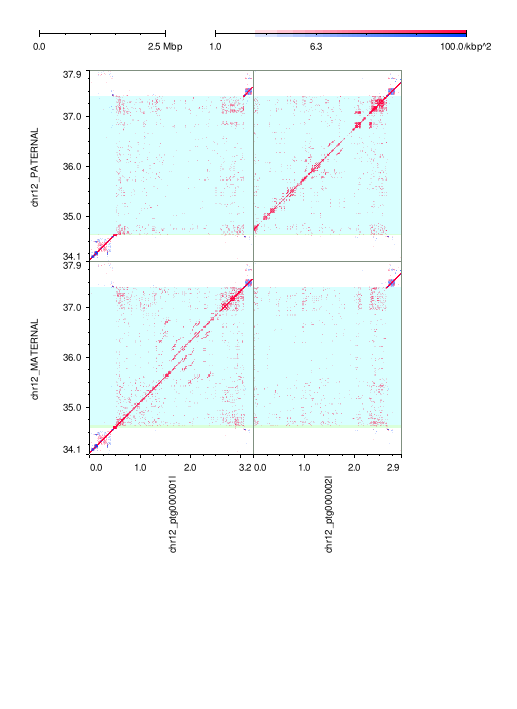

Supplement: btaf648_Supplementary_Data [file btaf648_supplementary_data.zip › Supplementary_Data/supplementary_figures/sample_matched_reference/chr12/hifiasm-ont-r1.png]

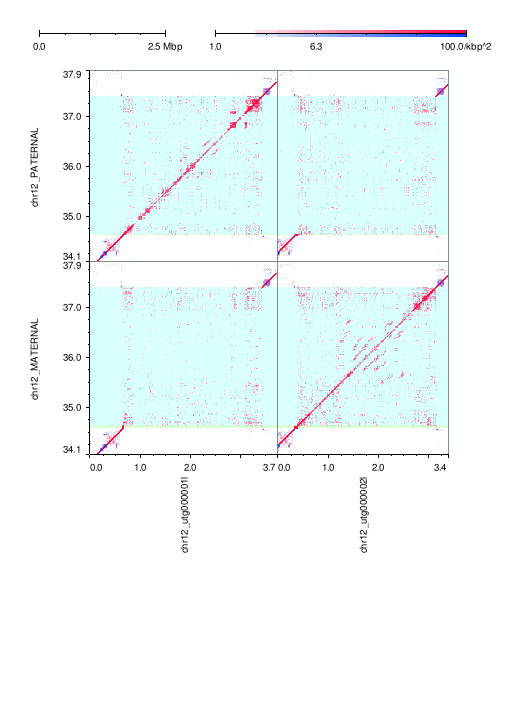

Supplement: btaf648_Supplementary_Data [file btaf648_supplementary_data.zip › Supplementary_Data/supplementary_figures/sample_matched_reference/chr12/mm2-ivh-hpc.png]

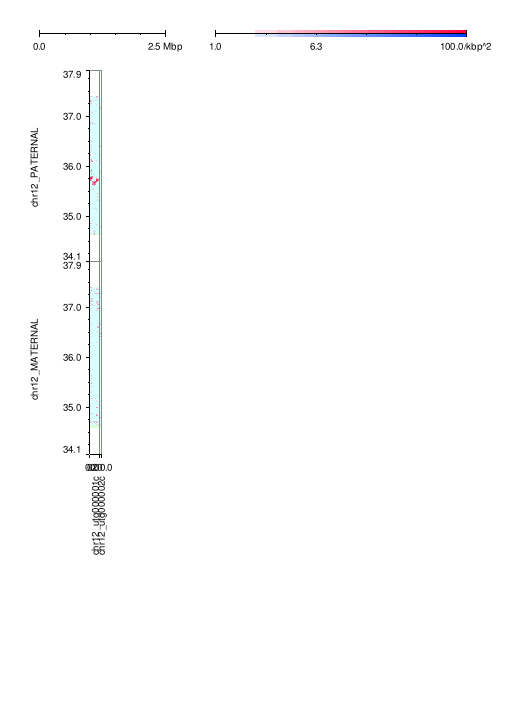

Supplement: btaf648_Supplementary_Data [file btaf648_supplementary_data.zip › Supplementary_Data/supplementary_figures/sample_matched_reference/chr12/mm2-k133-hpc.png]

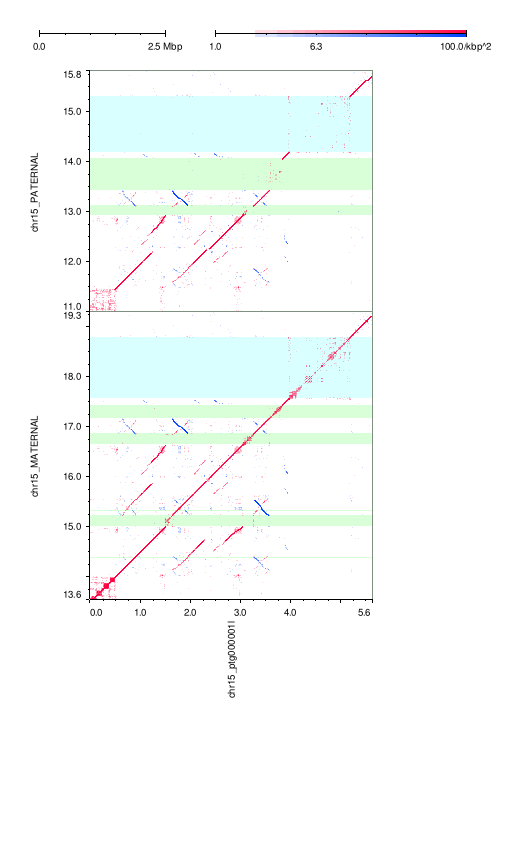

Supplement: btaf648_Supplementary_Data [file btaf648_supplementary_data.zip › Supplementary_Data/supplementary_figures/sample_matched_reference/chr15/hifiasm-ont-r1.png]

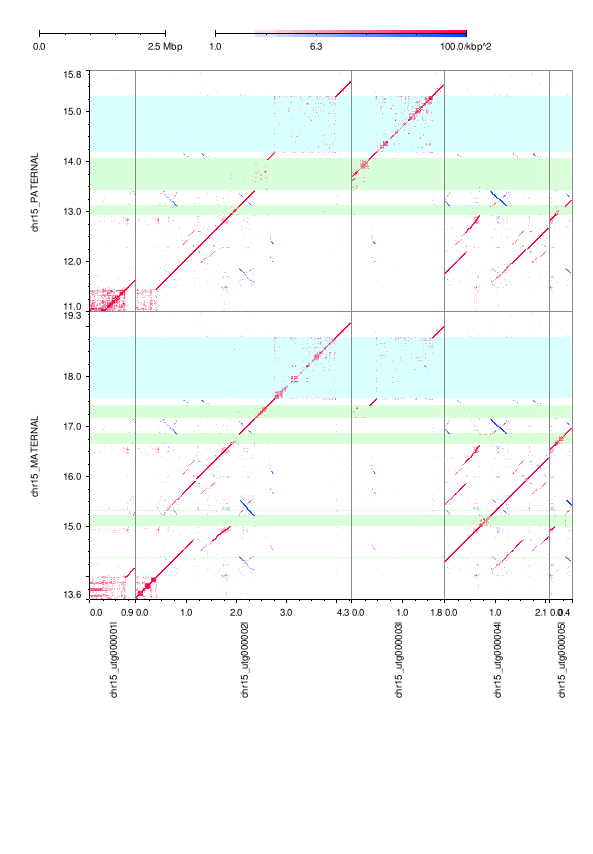

Supplement: btaf648_Supplementary_Data [file btaf648_supplementary_data.zip › Supplementary_Data/supplementary_figures/sample_matched_reference/chr15/mm2-ivh-hpc.png]

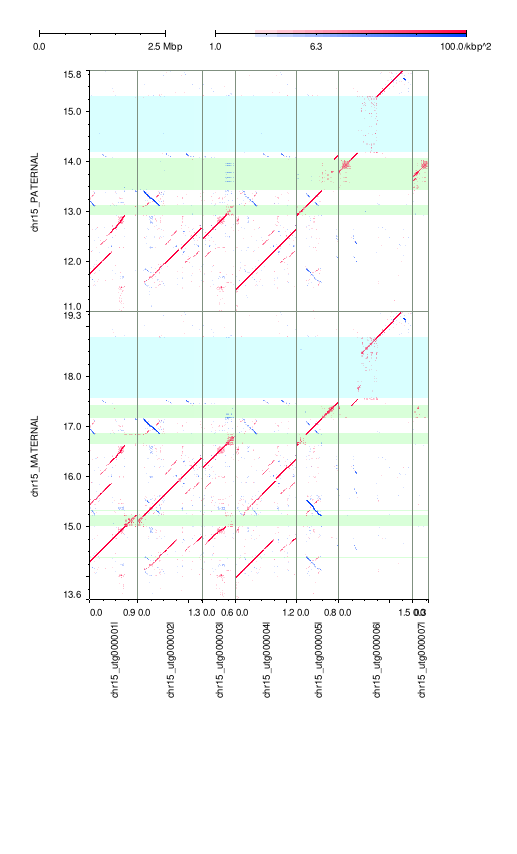

Supplement: btaf648_Supplementary_Data [file btaf648_supplementary_data.zip › Supplementary_Data/supplementary_figures/sample_matched_reference/chr15/mm2-k133-hpc.png]

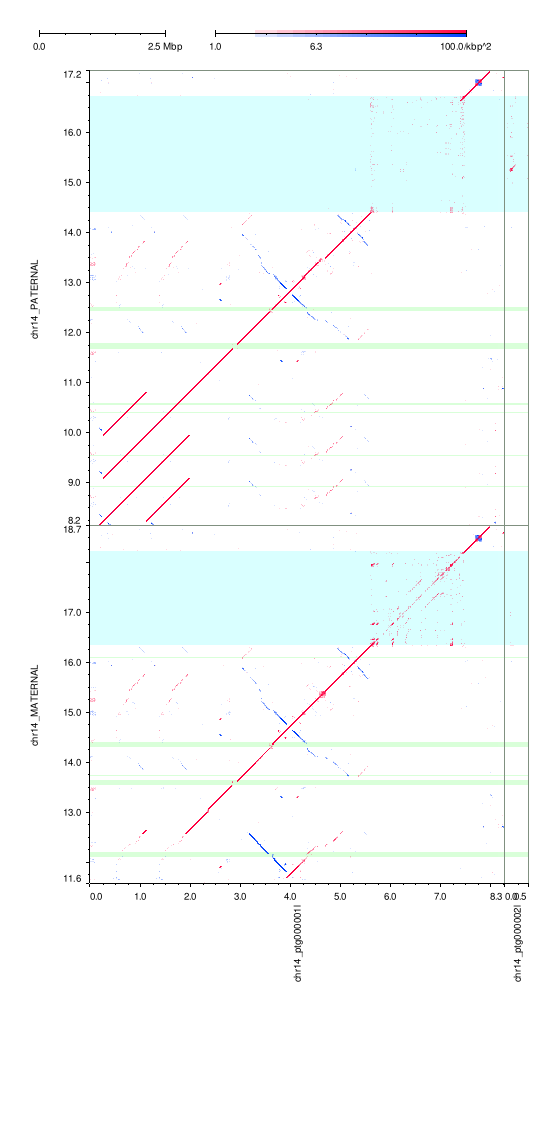

Supplement: btaf648_Supplementary_Data [file btaf648_supplementary_data.zip › Supplementary_Data/supplementary_figures/sample_matched_reference/chr14/hifiasm-ont-r1.png]

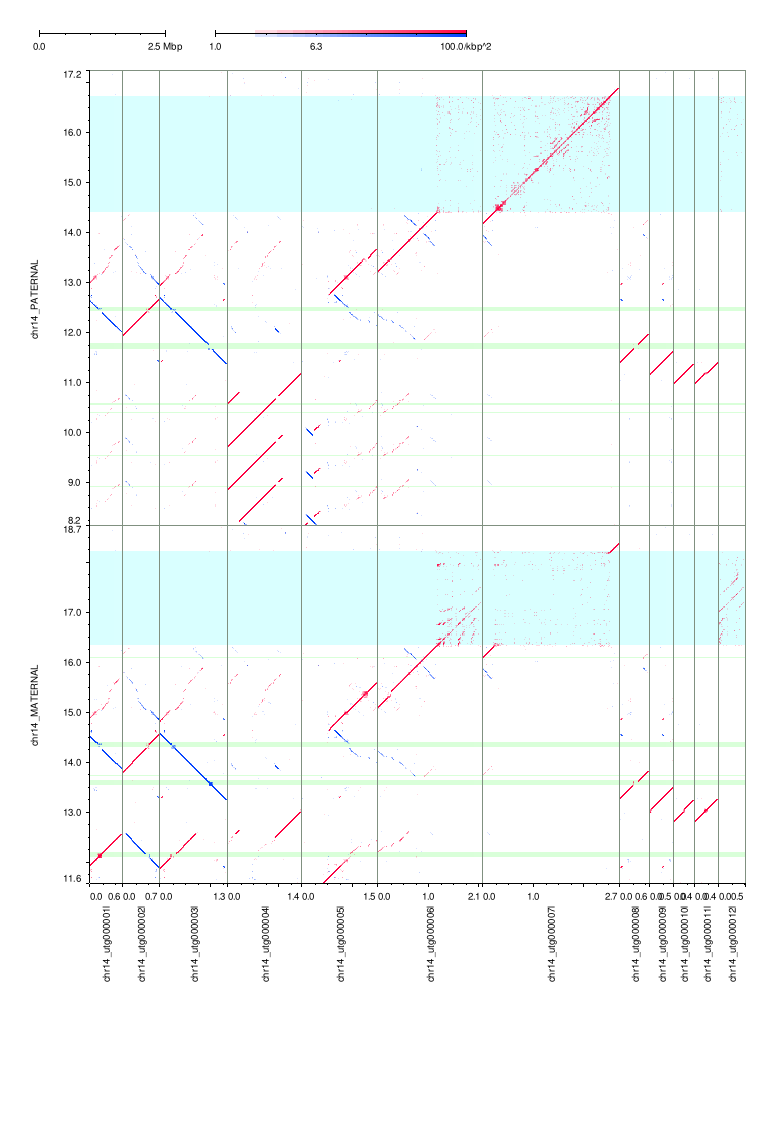

Supplement: btaf648_Supplementary_Data [file btaf648_supplementary_data.zip › Supplementary_Data/supplementary_figures/sample_matched_reference/chr14/mm2-ivh-hpc.png]

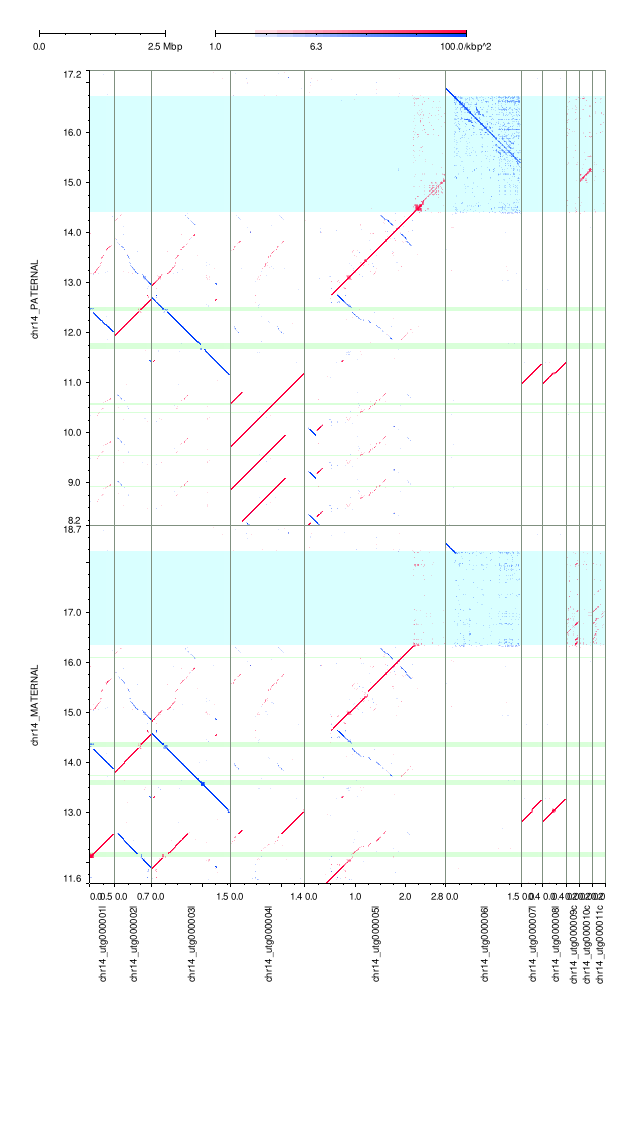

Supplement: btaf648_Supplementary_Data [file btaf648_supplementary_data.zip › Supplementary_Data/supplementary_figures/sample_matched_reference/chr14/mm2-k133-hpc.png]

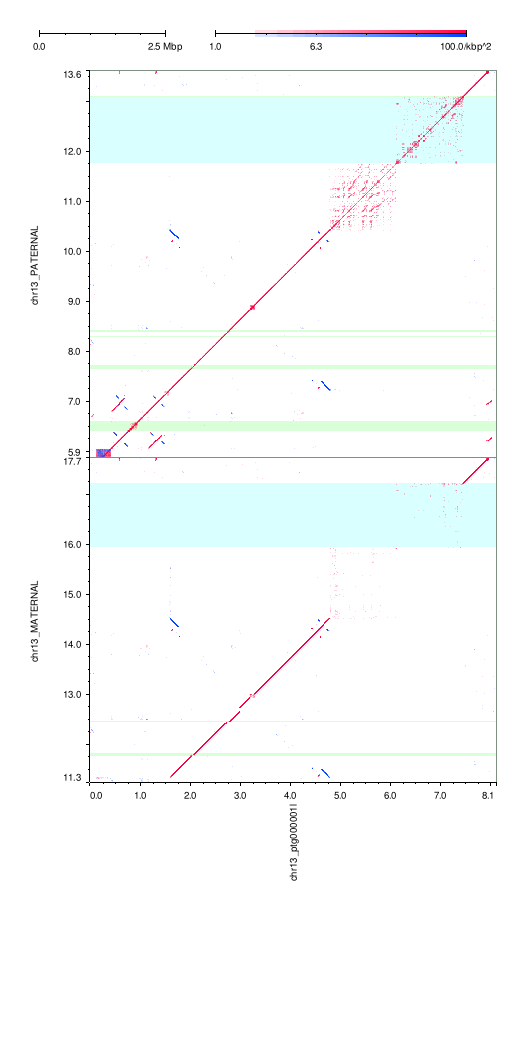

Supplement: btaf648_Supplementary_Data [file btaf648_supplementary_data.zip › Supplementary_Data/supplementary_figures/sample_matched_reference/chr13/hifiasm-ont-r1.png]

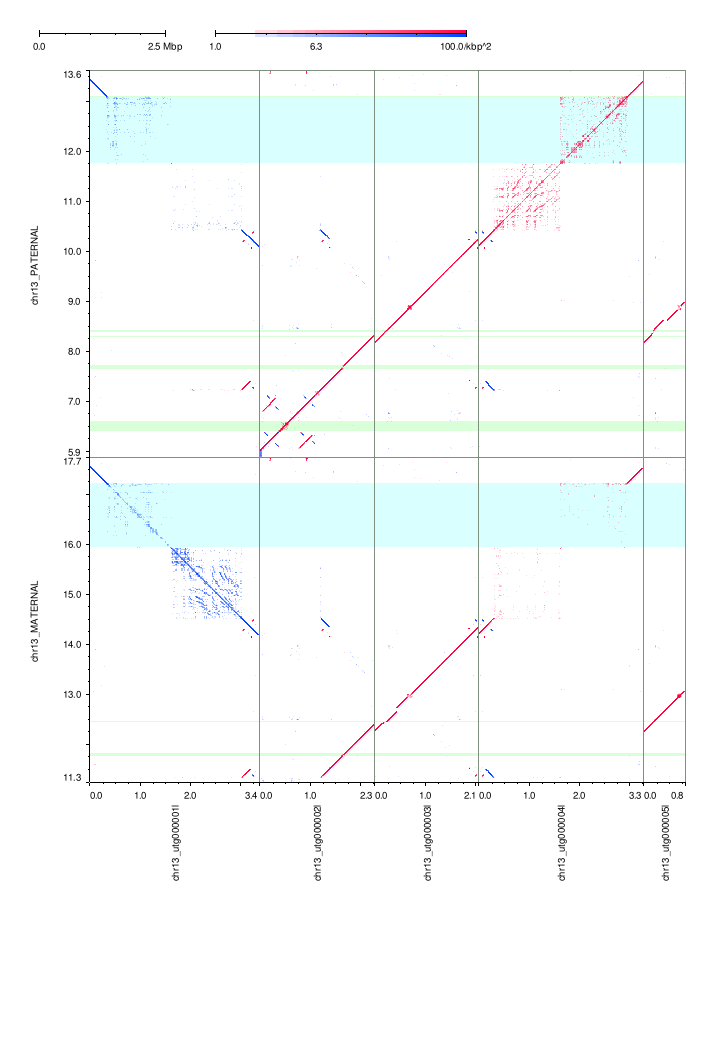

Supplement: btaf648_Supplementary_Data [file btaf648_supplementary_data.zip › Supplementary_Data/supplementary_figures/sample_matched_reference/chr13/mm2-ivh-hpc.png]

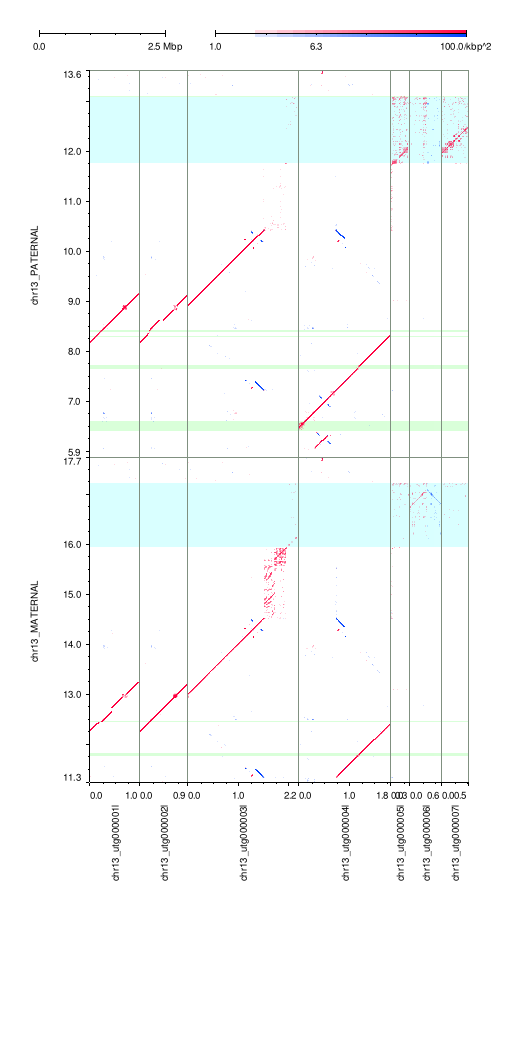

Supplement: btaf648_Supplementary_Data [file btaf648_supplementary_data.zip › Supplementary_Data/supplementary_figures/sample_matched_reference/chr13/mm2-k133-hpc.png]

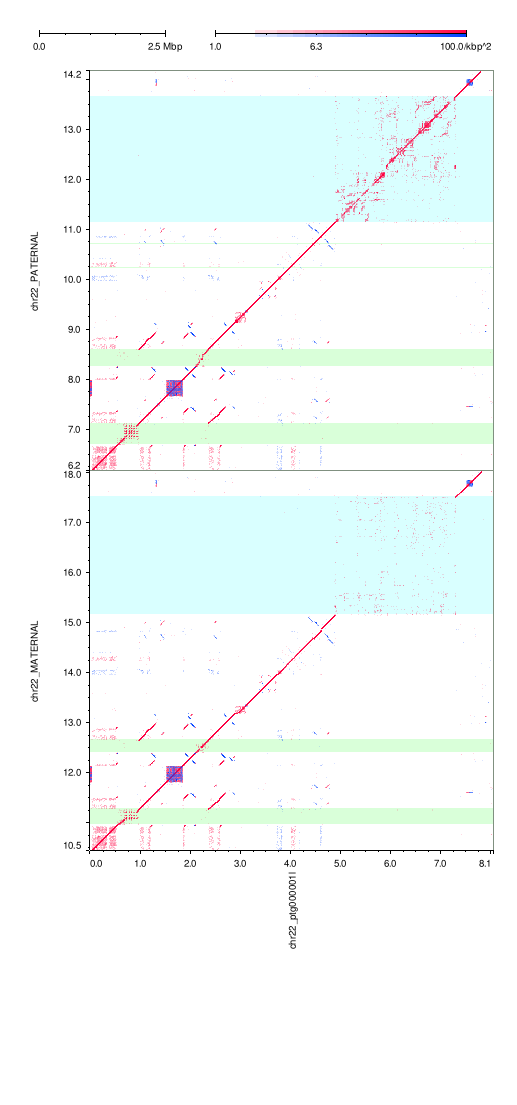

Supplement: btaf648_Supplementary_Data [file btaf648_supplementary_data.zip › Supplementary_Data/supplementary_figures/sample_matched_reference/chr22/hifiasm-ont-r1.png]

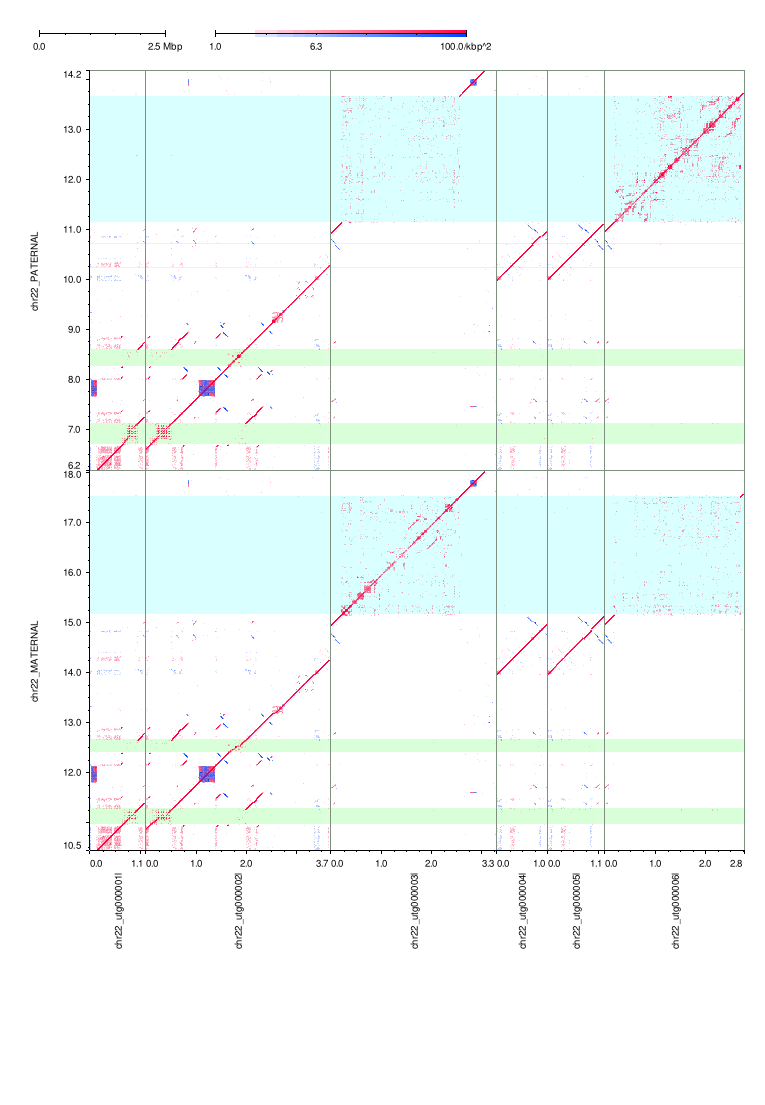

Supplement: btaf648_Supplementary_Data [file btaf648_supplementary_data.zip › Supplementary_Data/supplementary_figures/sample_matched_reference/chr22/mm2-ivh-hpc.png]

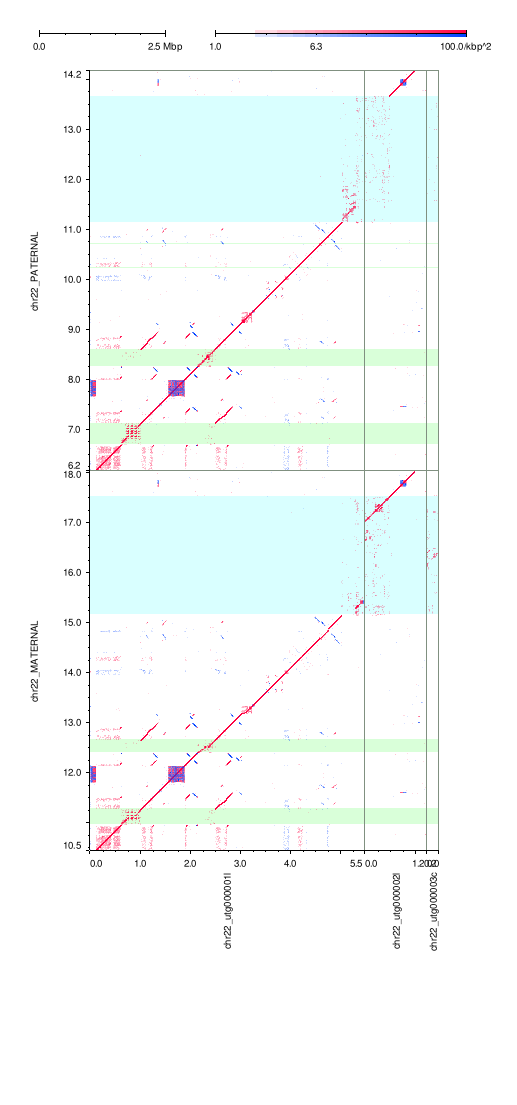

Supplement: btaf648_Supplementary_Data [file btaf648_supplementary_data.zip › Supplementary_Data/supplementary_figures/sample_matched_reference/chr22/mm2-k133-hpc.png]

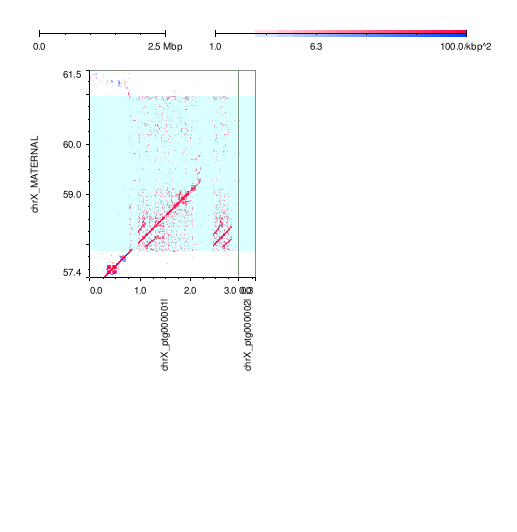

Supplement: btaf648_Supplementary_Data [file btaf648_supplementary_data.zip › Supplementary_Data/supplementary_figures/sample_matched_reference/chrX/hifiasm-ont-r1.png]

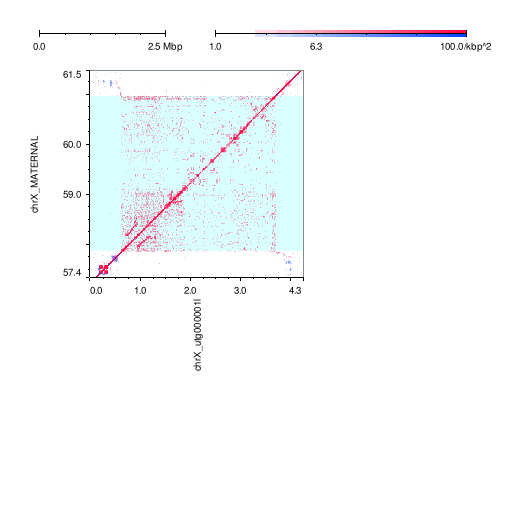

Supplement: btaf648_Supplementary_Data [file btaf648_supplementary_data.zip › Supplementary_Data/supplementary_figures/sample_matched_reference/chrX/mm2-ivh-hpc.png]

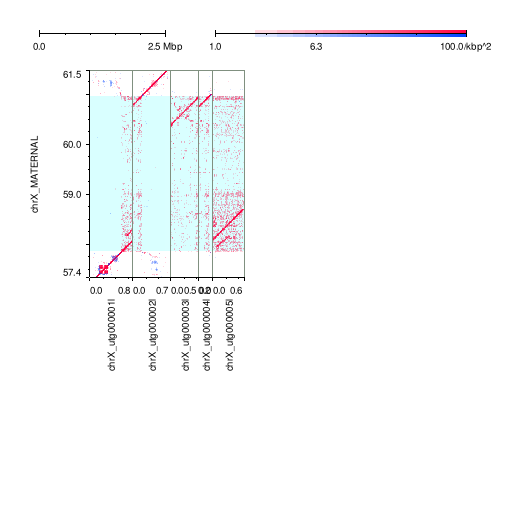

Supplement: btaf648_Supplementary_Data [file btaf648_supplementary_data.zip › Supplementary_Data/supplementary_figures/sample_matched_reference/chrX/mm2-k133-hpc.png]

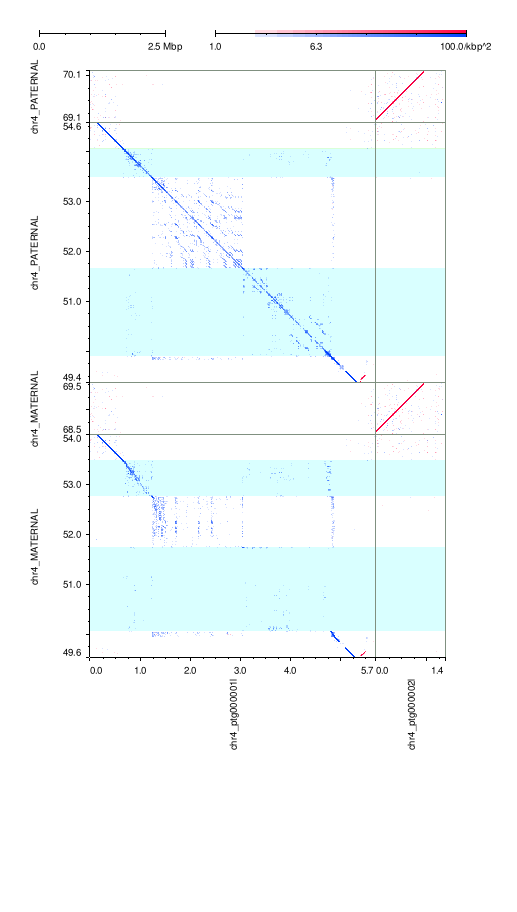

Supplement: btaf648_Supplementary_Data [file btaf648_supplementary_data.zip › Supplementary_Data/supplementary_figures/sample_matched_reference/chr4/hifiasm-ont-r1.png]

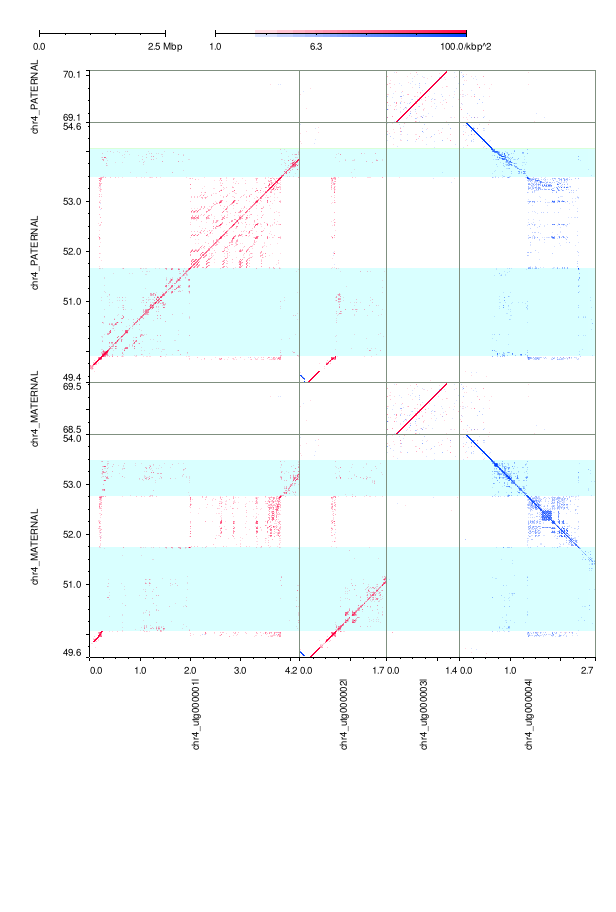

Supplement: btaf648_Supplementary_Data [file btaf648_supplementary_data.zip › Supplementary_Data/supplementary_figures/sample_matched_reference/chr4/mm2-ivh-hpc.png]

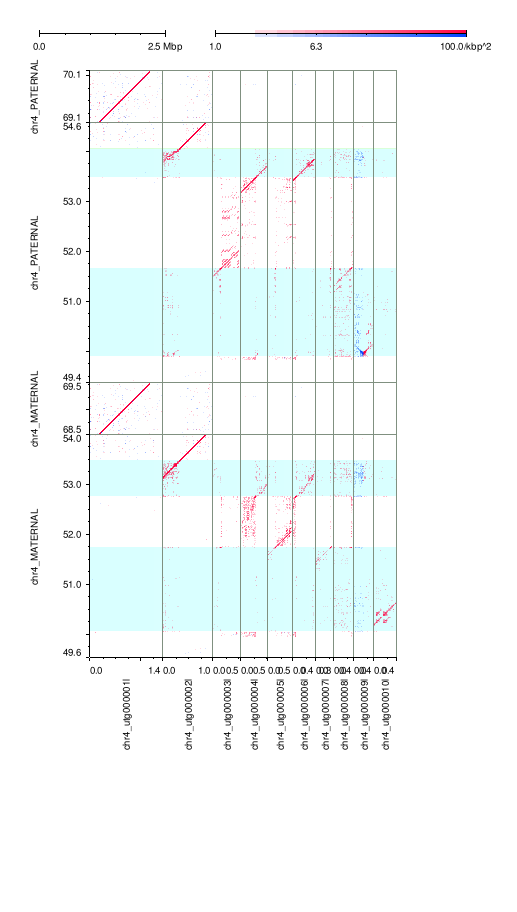

Supplement: btaf648_Supplementary_Data [file btaf648_supplementary_data.zip › Supplementary_Data/supplementary_figures/sample_matched_reference/chr4/mm2-k133-hpc.png]

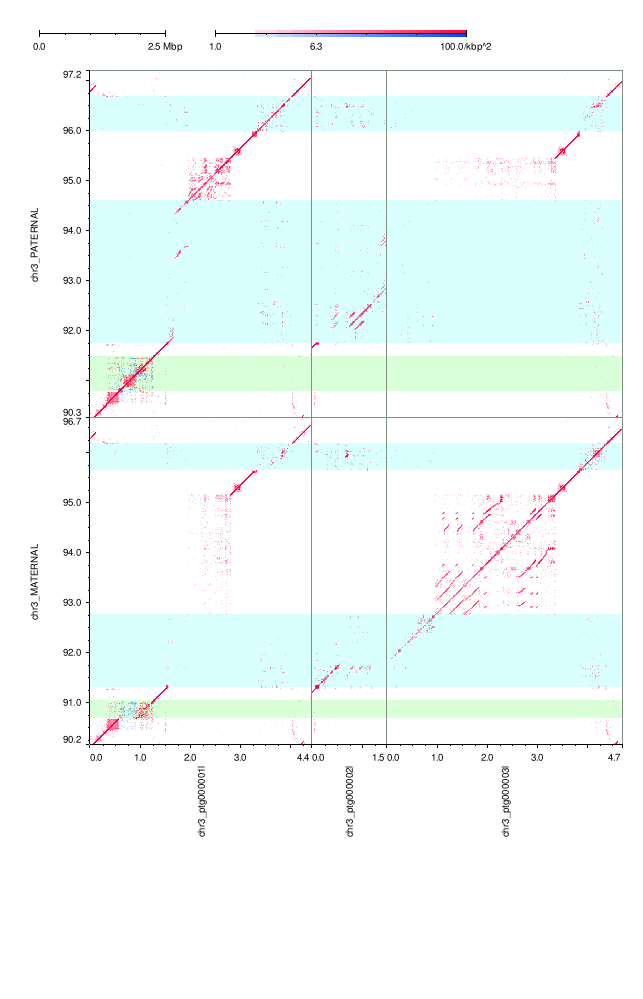

Supplement: btaf648_Supplementary_Data [file btaf648_supplementary_data.zip › Supplementary_Data/supplementary_figures/sample_matched_reference/chr3/hifiasm-ont-r1.png]

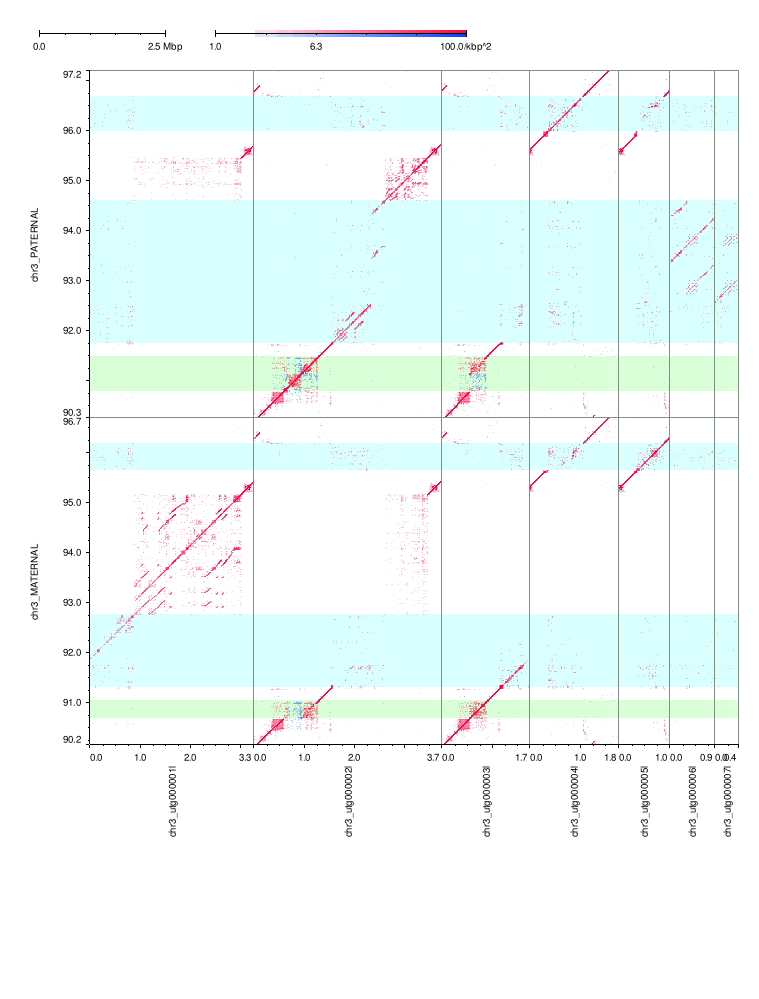

Supplement: btaf648_Supplementary_Data [file btaf648_supplementary_data.zip › Supplementary_Data/supplementary_figures/sample_matched_reference/chr3/mm2-ivh-hpc.png]

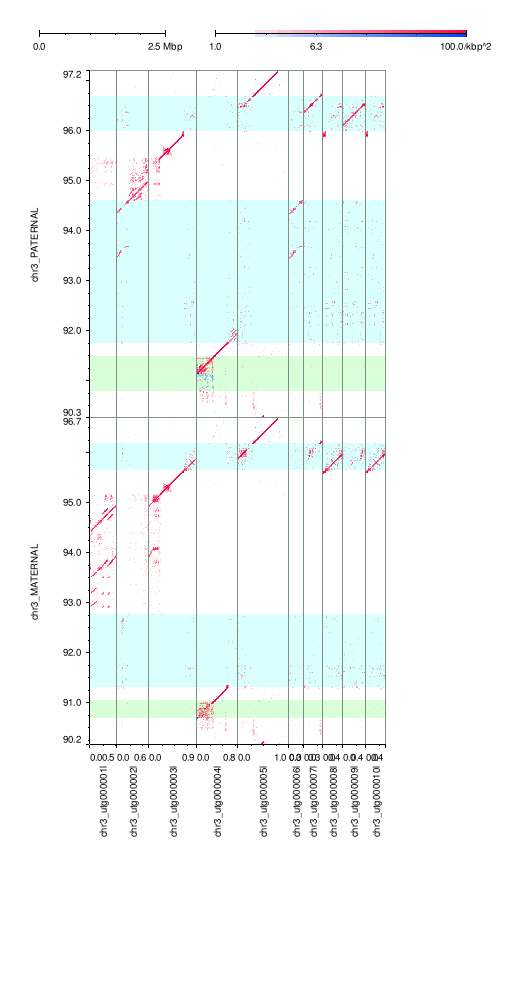

Supplement: btaf648_Supplementary_Data [file btaf648_supplementary_data.zip › Supplementary_Data/supplementary_figures/sample_matched_reference/chr3/mm2-k133-hpc.png]

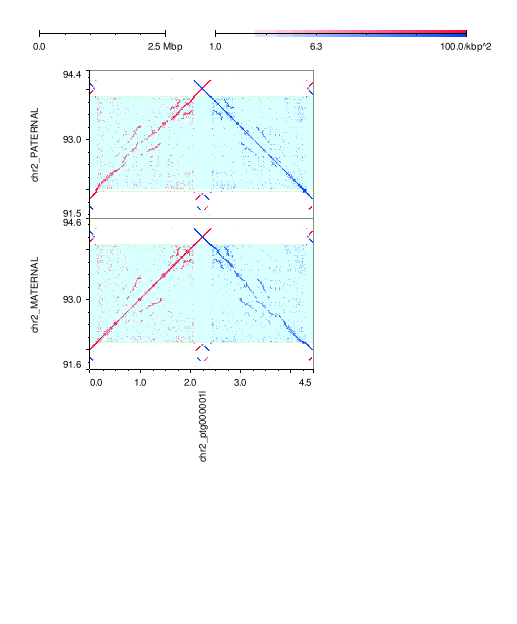

Supplement: btaf648_Supplementary_Data [file btaf648_supplementary_data.zip › Supplementary_Data/supplementary_figures/sample_matched_reference/chr2/hifiasm-ont-r1.png]

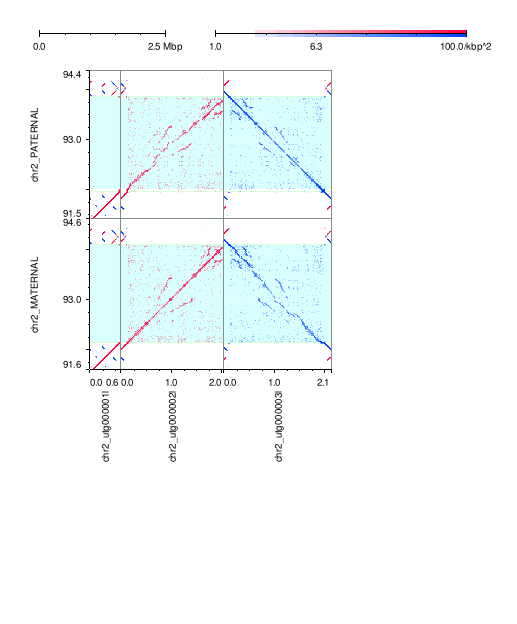

Supplement: btaf648_Supplementary_Data [file btaf648_supplementary_data.zip › Supplementary_Data/supplementary_figures/sample_matched_reference/chr2/mm2-ivh-hpc.png]

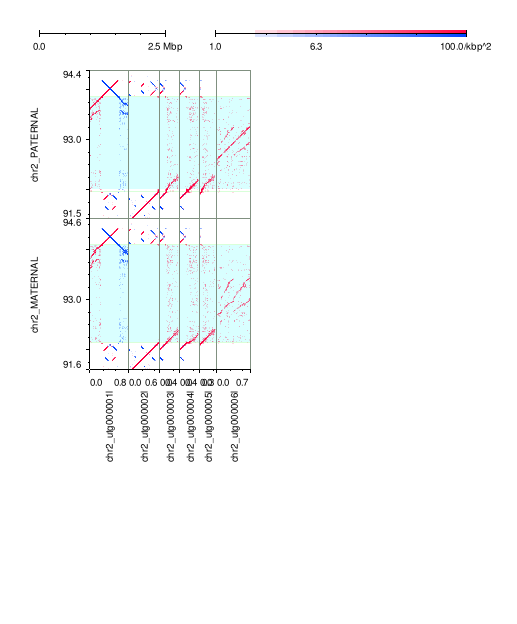

Supplement: btaf648_Supplementary_Data [file btaf648_supplementary_data.zip › Supplementary_Data/supplementary_figures/sample_matched_reference/chr2/mm2-k133-hpc.png]

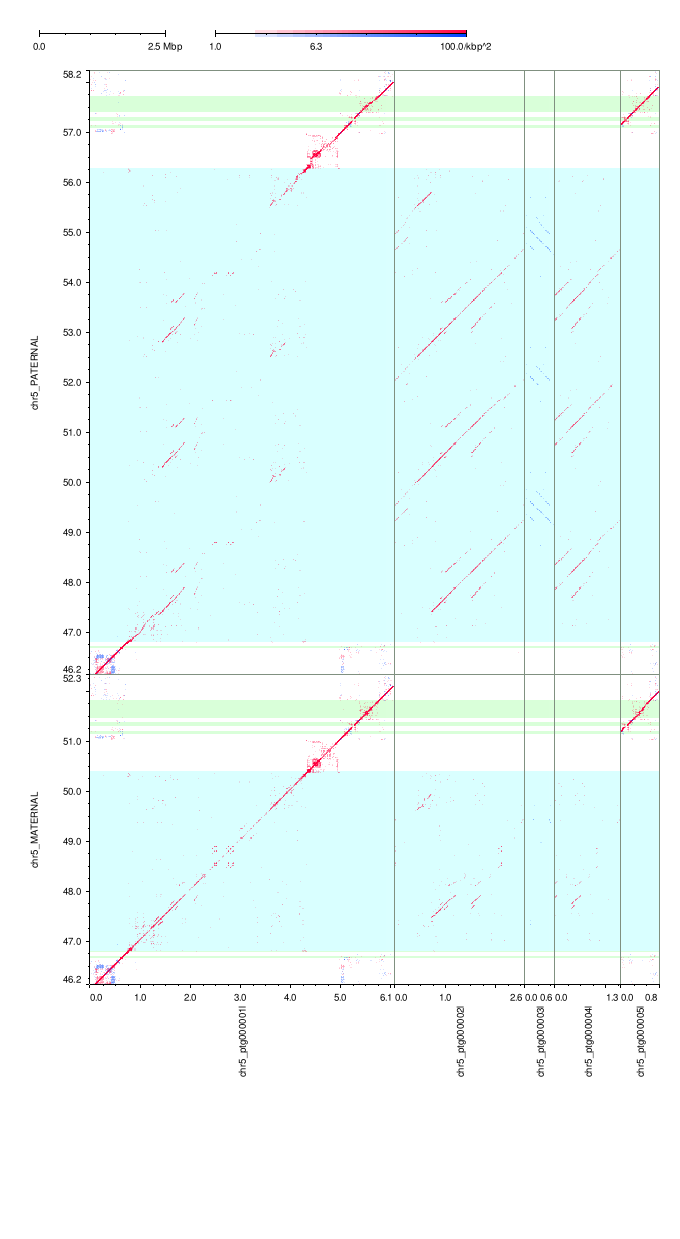

Supplement: btaf648_Supplementary_Data [file btaf648_supplementary_data.zip › Supplementary_Data/supplementary_figures/sample_matched_reference/chr5/hifiasm-ont-r1.png]
